# Outer Membrane Vesicles as Systems-Level Drivers of Neuroinflammation, Metabolic Dysfunction, and Proteinopathy in Alzheimer’s Disease

**DOI:** 10.3390/cells15080690

**Published:** 2026-04-14

**Authors:** Ali Delbaz, James A. St John

**Affiliations:** Clem Jones Centre for Neurobiology and Stem Cell Research, Institute for Biomedicine and Glycomics, Griffith University, Parklands Drive, Gold Coast, QLD 4222, Australia

**Keywords:** microbial extracellular vesicles, neuroimmune metabolism, olfactory neuroinvasion, glial bioenergetic stress

## Abstract

Alzheimer’s disease is a complex neurodegenerative condition characterized by progressive cognitive decline, neuroinflammation, metabolic dysregulation, and abnormal protein deposition. While genetic factors and amyloid-beta-focused hypotheses have been extensively investigated, they fail to fully account for the prolonged prodromal phase or the early susceptibility of olfactory and limbic regions. Emerging evidence suggests chronic peripheral and mucosal infections may influence disease risk; however, mechanisms by which microbial activity outside the central nervous system contributes to persistent neuropathology remain poorly understood. This review explores the emerging concept that bacterial outer membrane vesicles act as mobile, lipid-rich vectors linking peripheral microbial reservoirs to neuroimmune and metabolic dysfunction in the aging brain. We discuss evidence suggesting vesicles originating from oral, olfactory, and upper airway niches can access the central nervous system via vascular routes and direct neural pathways, including olfactory and trigeminal nerves, where they influence glial and endothelial cell function. We also propose the Accumulative Vesicle Load Hypothesis, which describes how cumulative lifetime exposure to bacterial vesicles shapes disease onset, anatomical vulnerability, and progression, and incorporates components of other hypotheses proposed for Alzheimer’s disease. This offers a system-level perspective for early diagnosis and upstream therapeutic strategies, including minimally invasive vesicle profiling in nasal fluid, saliva, blood, and cerebrospinal fluid. This work is a conceptual review that summarizes current evidence in a hierarchically organized manner and proposes a testable model; it does not assert causality where direct human evidence is currently limited.

## 1. Introduction

### 1.1. Association Between Infection Risk and Alzheimer’s Disease

Alzheimer’s disease (AD) is a progressive neurodegenerative condition marked by the accumulation of amyloid-β (Aβ) plaques, synaptic degeneration, widespread neuroinflammation, and neurofibrillary tangles composed of hyperphosphorylated tau [1]. Although genetic risk factors such as the APOE4 allele and disruptions in Aβ processing pathways have been extensively characterized, these mechanisms alone do not fully account for the persistent inflammatory state and metabolic dysfunction observed in AD brains [2,3,4]. Emerging epidemiological and experimental evidence suggests chronic peripheral and mucosal infections may act as significant modulators of AD risk and progression [5,6,7]. This is known as the emerging infectious disease hypothesis of AD. Nevertheless, precise biological pathways through which microbial signals originating outside the central nervous system (CNS) exert sustained influence on brain pathology remain poorly understood.

In parallel with the emerging infectious hypothesis of AD, glial cells, particularly microglia and astrocytes, have become central to understanding the disease pathophysiology. Disease-associated microglia and reactive astrocytes undergo profound metabolic remodeling, inflammatory reprogramming, and loss of neuro-supportive functions. These glial alterations directly contribute to synaptic dysfunction, neuronal energy failure, and accelerated protein aggregation [8,9,10]. Notably, many of these glial phenotypes resemble responses typically induced by bacterial components such as lipopolysaccharide (LPS), peptidoglycan, and microbial proteases [11,12,13,14,15]. This observation raises a critical mechanistic question: how do microbial signals originating outside the CNS reach and chronically activate glial cells in the aging brain?

Outer membrane vesicles (OMVs) have recently emerged as a biologically plausible and highly potent mechanism to address this question [16,17,18]. OMVs are nanoscale, lipid-rich vesicles that are constitutively released by Gram-negative bacteria and carry a concentrated array of virulence factors, including LPS, membrane proteins, toxins, enzymes, and bacterial nucleic acids [19,20,21]. Unlike free bacterial components, OMVs possess structural integrity that confers protection, enables long-range transport, and facilitates efficient uptake by host cells through lipid raft-mediated endocytosis and macropinocytosis [22,23,24]. This unique combination of stability, mobility, and immunogenic cargo positions OMVs as ideal vectors for sustained delivery of microbial signals from peripheral mucosal environments to the CNS. While OMVs are produced by a broad range of Gram-negative bacteria [22,23,24], this review focuses specifically on airway, oral, and neurotropic pathobionts with established or emerging relevance to AD. These include *Moraxella catarrhalis*, *Haemophilus influenzae*, and *Neisseria meningitidis* from the upper respiratory tract and olfactory interface, as well as the periodontal pathogen *Porphyromonas gingivalis* from the oral–systemic axis [25,26,27,28,29]. In addition, vesicle-like extracellular structures and chronic intracellular infection by *Chlamydia pneumoniae* are considered, given their strong experimental links to glial infection, neuroinflammation, and AD-related pathology [30,31].

This manuscript is written as a conceptual review that integrates direct human evidence, mechanistic infection-biology studies, and broader extracellular-vesicle research within a structured interpretive framework. It is not presented as a hypothesis article; rather, it synthesizes existing findings and organizes them into a testable model, the Accumulative Vesicle Load Hypothesis. To maintain conceptual clarity, the review separates Tier A evidence from AD cohorts, Tier B infection-biology and CNS-relevant vesicle mechanisms, and Tier C extrapolations from general extracellular-vesicle and neuroinflammation studies. This structure enables a balanced and transparent evaluation of current data while outlining mechanistic trajectories that remain open for empirical validation.

Throughout, we indicate the experimental context for each quantitative result as human cohort, human primary or immortalized cells, animal model, or mixed in vitro co-culture, to aid interpretation of diagnostic performance and mechanistic strength.

Scope and definitions. In this review, outer membrane vesicles (OMVs) refer only to nanovesicles shed from the outer membrane of Gram-negative bacteria. Vesicular entities from other sources are mentioned comparatively and are not labeled OMVs. These include Vesicle-like extracellular structures formed by intracellular bacteria such as *Chlamydia pneumoniae*, and host-derived extracellular vesicles (exosomes and microvesicles). The title, figures, and main text follow this usage, and causal claims are limited to OMVs unless explicitly stated otherwise.

To align claims with available evidence, we use cautious formulations such as “biomarker candidates,” “therapeutic candidates,” “plausible route,” and “plausible contributors,” we explicitly note that direct OMV detection in early human AD remains limited.

### 1.2. Early Anatomical Progression of AD

AD follows a highly ordered anatomical trajectory. Pathology begins within olfactory and limbic structures, including the olfactory bulb, piriform cortex, and entorhinal cortex, before progressing into the hippocampus and associative cortical regions [32]. Early olfactory dysfunction is one of the most reliable prodromal markers and often appears years before cognitive decline [33]. The entorhinal cortex and hippocampus subsequently develop synaptic dysfunction, glial activation, and early Aβ and tau pathology, ultimately leading to the widespread cortical degeneration observed in later stages. This ordered progression provides a biological rationale for investigating peripheral–central pathways that could selectively affect these vulnerable regions [32].

Quantitatively, odor identification performance demonstrates meaningful predictive utility for cognitive decline and incident AD in community cohorts. The University of Pennsylvania Smell Identification Test (UPSIT), a standardized 40-item odor identification measure widely used in clinical and research contexts, provides one such index. In a longitudinal study of 1037 adults without dementia, each one-point lower baseline UPSIT score was associated with a seven to ten percent higher hazard of transition to AD during follow-up (hazard ratio per point 1.072 to 1.099), independent of demographic variables and baseline cognitive performance. Meta-analytic evidence further indicates that olfactory testing discriminates AD with a pooled sensitivity of approximately 0.79 and specificity of 0.78, and identifies mild cognitive impairment with sensitivity of 0.67 and specificity near 0.79 across twenty-five studies [34].

### 1.3. Evolutionary Vulnerability of the Olfactory System

From an evolutionary perspective, the olfactory system represents a unique neurobiological balance between rapid environmental sensing and immune isolation. Unlike other sensory systems, the olfactory neuroepithelium maintains direct axonal continuity with limbic brain regions, reflecting its ancient role in detecting food, predators, toxins, and social cues essential for survival [35,36]. This architecture evolved long before the advent of extended human lifespans, modern urbanization, or chronic inflammatory diseases, and therefore prioritized speed and sensitivity over long-term immune containment [37,38].

OMVs exploit this evolutionary vulnerability. Their nanoscale dimensions, lipid-rich membranes, and resistance to degradation enable them to traverse anatomical interfaces that evolved to handle transient microbial exposure rather than a lifelong vesicular burden. In ancestral environments, episodic infections and shorter lifespans likely limited cumulative vesicle exposure [16,18]. In contrast, modern humans experience decades of repeated airway colonization, chronic rhinosinusitis, periodontal disease, viral–bacterial co-infections, air pollution, and smoking-related epithelial injury, all of which increase bacterial vesiculation [16,18,25].

This evolutionary mismatch provides a compelling explanation for why olfactory and limbic circuits, among the most evolutionarily conserved brain regions, are also among the earliest and most vulnerable in AD [35,39]. Rather than being accidental targets, these regions occupy privileged positions at the interface between microbial ecosystems and the CNS, rendering them uniquely sensitive to chronic vesicle-mediated immune and metabolic stress [35,37].

### 1.4. The Olfactory Nerve Pathway as an Early Anatomical Conduit for AD-Related Pathology

The earliest pathological changes in AD consistently occur within olfactory–limbic circuits, including the olfactory bulb, piriform cortex, entorhinal cortex, and hippocampus [40]. These regions show early synaptic impairment, glial activation, and increased vulnerability to inflammatory and metabolic stressors long before widespread cortical involvement becomes apparent. This anatomical sequence strongly suggests that disease-relevant signals may enter or influence the brain through structures that are directly connected to the external environment. Among these, the olfactory epithelium and its associated neuronal pathways represent the most direct and evolutionary vulnerable interface. Understanding how peripheral signals access these early-affected regions provides essential context for later sections addressing vesicle trafficking and systems-level vulnerability [32].

**Figure 1:**
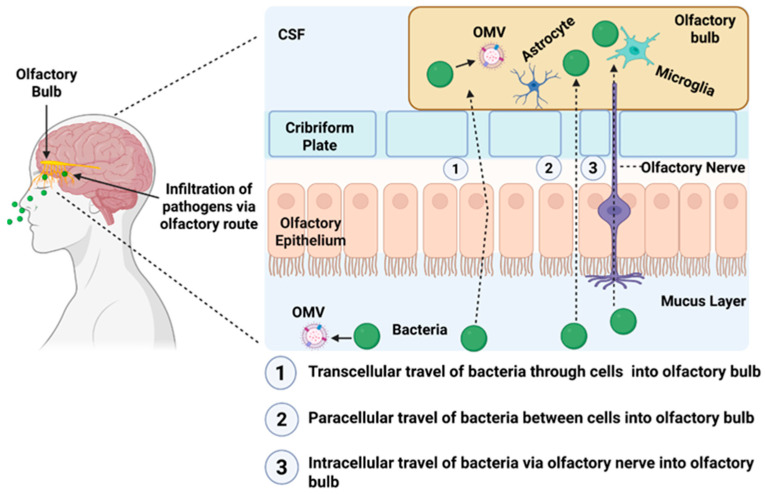
Olfactory epithelial and neuronal pathways enabling vesicular access to the brain. Schematic illustrating proposed mechanisms by which microbial products and associated OMVs originating in the nasal cavity may access the olfactory bulb. Following deposition within the mucus layer, vesicles and microbial components may traverse the olfactory epithelium via (1) transcellular transport through epithelial cells, (2) paracellular passage between cells following junctional disruption, or (3) intracellular trafficking along unmyelinated axons of olfactory sensory neurons projecting through the cribriform plate. These routes allow vesicle-associated cargo to bypass classical blood–brain barrier constraints and directly access the olfactory bulb, where early interactions with astrocytes, microglia, and olfactory ensheathing cells may initiate neuroinflammatory and metabolic responses. The schematic focuses on the olfactory route; trigeminal sensory projections represent a parallel neuronal pathway that is not depicted here. Although intact bacteria are depicted for anatomical clarity, OMVs released from these organisms are proposed to traffic along the same epithelial and neuronal pathways. Created in BioRender. Mathiske, T. (2026) https://Biorender.com/iail9tw.

Among all potential routes, the olfactory nerve pathway offers the most direct and barrier-independent conduit to the brain. As illustrated schematically (Figure 1), microbial products and their associated OMVs may traverse the olfactory epithelium via transcellular, paracellular, or intracellular routes before accessing the olfactory bulb. The olfactory neuroepithelium is uniquely exposed to external environments and is separated from the brain only by the cribriform plate [41]. Unmyelinated axons of olfactory sensory neurons project directly from the nasal cavity into the olfactory bulb, creating an anatomical vulnerability that nanoscale microbial vesicles can exploit. Experimental work has confirmed this, demonstrating that vesicular particles and microbial components deposited intranasally can undergo axonal transport into the olfactory bulb within hours to days, providing a plausible route for peripheral microorganism signals to access the CNS [42,43,44]. Once there, vesicles encounter a dense network of olfactory ensheathing cells (OECs), astrocytes and microglia, all of which display high endocytic capacity and robust innate immune reactivity [45,46].

The nasal–olfactory–brain axis, therefore, provides a coherent mechanistic explanation for two long-standing clinical observations. First, olfactory dysfunction is one of the earliest prodromal features of AD and often precedes cognitive decline by many years [47]. Second, the composition of nasal and upper airway microbiota is altered in individuals at risk of neurodegeneration, suggesting sustained exposure of the olfactory system to microbial products initiates disease processes [35,43]. Within this framework, OMVs serve as highly efficient neuroinvasive vectors that bypass systemic immune clearance and deliver concentrated microbial cargo directly into vulnerable brain entry points [43].

## 2. OMVs as Unifying Mechanistic Links

Traditional models of infection-associated neurodegeneration have largely focused on two pathways: direct invasion of the CNS by whole bacteria or viruses, or passive diffusion of soluble bacterial toxins across the blood–brain barrier (BBB) [48]. However, neither mechanism adequately explains the chronic, low-grade, and spatially heterogeneous neuroinflammation that characterizes AD. In most circumstances, whole-cell invasion tends to be rare and acute, and free bacterial toxins are rapidly degraded or neutralized in circulation [16,49,50]. In contrast, OMVs represent a third and more insidious biological strategy that enables continuous, protected, and multimodal delivery of microbial signals to CNS barriers and resident glial cells [14,17].

OMVs have been shown to cross epithelial barriers, enter systemic circulation, and interact directly with endothelial cells of the BBB to compromise tight junction integrity and facilitate transcytosis [51,52,53]. Of particular relevance to AD, OMVs from bacteria such as *Porphyromonas gingivalis*, a periodontal pathogen strongly implicated in AD, can gain direct access to the brain via the olfactory nerve pathway, bypassing the BBB entirely [54,55]. Thus, the olfactory neuroepithelium represents one of the most vulnerable neuroimmune interfaces in humans, and early olfactory dysfunction is among the most consistent prodromal features of AD. Within this context, OMV trafficking from nasal and oral microbiota to the olfactory bulb offers a compelling biological route through which chronic mucosal infection may initiate neuroinflammation years before cognitive symptom onset [24,41,56,57].

Once inside the CNS, OMVs exert profound effects on glial biology. Microglia respond to OMV cargo through activation of Toll-like receptors such as TLR2 and TLR4, NOD-like receptors, and inflammasome pathways which drives a shift toward pro-inflammatory, lipid-laden disease-associated phenotypes [17,58,59,60]. Astrocytes similarly undergo reactive transformation characterized by mitochondrial suppression, impaired glucose and lipid metabolism, and diminished neurotrophic support [61,62]. These glial changes are not transient; rather, they establish self-perpetuating inflammatory and metabolic feedback loops that amplify oxidative stress, disrupt neuron–glial coupling, and render neural networks increasingly vulnerable to degeneration [53,60,63,64,65]. In this way, OMVs function not as isolated inflammatory stimuli but as systems-level reprogramming agents that reshape immune and metabolic homeostasis within the CNS [53,66,67].

## 3. OMVs as Modulators of Neuroinflammation, Metabolism, and Proteinopathy

Beyond their immunostimulatory properties, OMVs exert profound effects on cellular metabolism, which is increasingly recognized as a hallmark of AD pathogenesis. Exposure to OMVs suppresses oxidative phosphorylation, promotes a glycolytic shift, disrupts β-oxidation of fatty acids, and induces the accumulation of toxic lipid intermediates such as ceramides [16,17,53,68]. These metabolic disturbances occur in both microglia and astrocytes and are further amplified under hypoxic conditions commonly observed in aging and AD-affected brains due to vascular dysfunction and cerebral hypoperfusion [24,67,69,70,71]. The convergence of OMV-driven inflammation, metabolic rewiring, and hypoxia creates a hostile bioenergetic environment that severely compromises neuronal resilience.

Critically, these OMV-induced metabolic and inflammatory states intersect directly with core proteinopathies of AD. OMVs from *Porphyromonas gingivalis* carry LPS and gingipains, which enhance amyloidogenic processing of amyloid precursor protein by clustering β-secretase within lipid rafts [55,72]. Virulence factors contained in OMVs from periodontal pathogens such as *Aggregatibacter actinomycetemcomitans* and *Fusobacterium nucleatum*—including LPS, outer membrane proteins, and gingipain-like proteases—activate pro-inflammatory and kinase signaling pathways (NF-κB, p38 MAPK, GSK-3β) [73,74,75]. *In vitro*, OMVs from *Fusobacterium nucleatum* have also been shown to suppress phosphorylation of these kinases in neuronal cells, pathways which are implicated in tau hyperphosphorylation and misfolding [55,76]. Additionally, OMVs from gut-associated bacteria like *Escherichia coli* contain lipid components and nucleic acids that amplify neuroinflammatory cascades, and emerging evidence suggests OMV surfaces themselves act as pathological nucleation platforms for Aβ aggregation, providing a scaffold for Aβ fibrillization. Collectively, these observations position OMVs not merely as upstream inflammatory stimuli but as integrative drivers that simultaneously promote Aβ accumulation, tau pathology, and glial-mediated neurotoxicity [77,78].

### 3.1. Bacterial Vesicle Heterogeneity and Pathogen-Specific Signatures

Although OMVs share core structural features, their biological impact is shaped by species-specific differences in cargo composition, lipid architecture, and regulatory pathways governing vesicle biogenesis. These differences generate distinct “vesicle fingerprints” that influence how individual bacterial reservoirs contribute to neuroinflammation, metabolic dysfunction, and proteinopathy [22,79,80].

Airway-associated pathobionts such as *Moraxella catarrhalis* release OMVs enriched in UspA1/UspA2 and Hag/MID adhesins that bind CEACAM receptors and IgD, promoting B-cell activation, chronic mucosal inflammation, and sustained innate immune priming [81,82,83]. Non-typeable *Haemophilus influenzae* produces OMVs containing P5 and P6 outer-membrane proteins and phosphorylcholine-modified Lipooligosaccharides (LOS). These engage TLR2, TLR4, and platelet-activating factor receptors, amplifying epithelial cytokine release and barrier permeability. *Neisseria meningitidis* OMVs are dominated by PorB and highly immunostimulatory LOS, potently activating TLR4–MD2, inflammasome pathways, and mitochondrial stress responses in myeloid cells [84,85,86,87]. Periodontal *Porphyromonas gingivalis* OMVs concentrate gingipain proteases, fimbriae, and atypical LPS lipid A structures that selectively engage TLR2 and complement receptor C5aR [80,88,89,90]. These unique signaling biases enable immune subversion rather than overt inflammation, driving chronic low-grade activation, and complementing dysregulation and kinase cascades linked to tau hyperphosphorylation. For intracellular pathogens such as *Chlamydia pneumoniae*, vesicle-like particles and host-derived exosomes enriched in bacterial antigens provide additional alternative routes for disseminating inflammatory signals beyond the primary infection site [91,92,93].

Importantly, despite these differences, vesicles from diverse bacterial species converge on a limited set of host outcomes: chronic glial activation, mitochondrial suppression, lipid dysregulation, and impaired protein clearance. This convergence suggests that vesicle format, rather than any single pathogen, is the dominant driver of Alzheimer’s-relevant pathology [53,94,95,96].

### 3.2. Synergistic Interaction of Hypoxia and Vesicle Exposure

Cerebral hypoperfusion and vascular insufficiency are common features of aging brains and early AD, and act as powerful amplifiers of OMV-driven pathology. Hypoxia stabilizes hypoxia-inducible factor-1α (HIF-1α), suppresses mitochondrial oxidative phosphorylation, and shifts glial metabolism toward glycolysis. These changes reduce metabolic flexibility and increase sensitivity to inflammatory stimuli [97,98,99,100]. In addition, hypoxia enhances TLR responsiveness and lowers the activation threshold for inflammasome signaling, such that a given vesicle burden produces disproportionately greater inflammatory output under hypoxic conditions [53,101]. Endothelial hypoxia further increases BBB permeability and vesicle transcytosis, creating a feed-forward loop between vascular dysfunction and vesicle entry [102,103]. OMVs then exacerbate hypoxia-driven vulnerability by impairing mitochondrial respiration, increasing reactive oxygen species (ROS), and disrupting NAD^+^ regeneration.

Together, hypoxia and OMVs form a two-hit system in which vascular compromise primes the brain for vesicle toxicity, while vesicles accelerate hypoxia-driven metabolic collapse. This synergy provides a mechanistic explanation for why vascular risk factors strongly interact with infection and inflammation to accelerate AD progression.

## 4. Peripheral to Central Transport of OMVs

**Figure 2:**
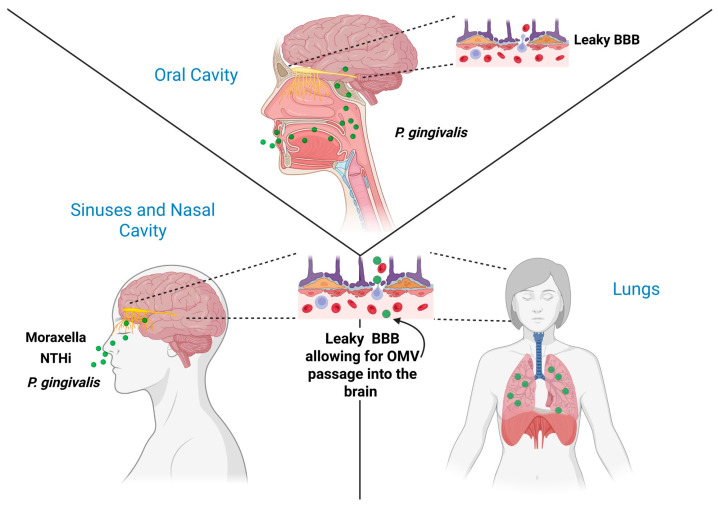
Peripheral epithelial and vascular pathways for OMV entry. This schematic illustrates how bacterial OMVs cross oral, nasal, and airway epithelial surfaces and enter systemic circulation, where they interact with the BBB. Neural pathways are not shown here and are illustrated separately in Figure 1. NTHi = non-typeable *Haemophilus influenzae*. Created in BioRender. Mathiske, T. (2026) https://BioRender.com/iail9tw.

A requirement for any infection-linked model of AD is a biologically plausible route by which microbial signals generated at peripheral or mucosal surfaces can reach the CNS in a chronic, sustained, and pathophysiologically meaningful manner (Figure 2). OMVs possess a distinctive set of biophysical properties that separate them from free bacterial toxins or whole organisms [19,104]. They are nanoscale (approximately 20 to 250 nm), lipid encapsulated, protease resistant, and optimized for cellular uptake and long-range trafficking. Together, these features allow OMVs to function as highly efficient communication vectors, which reach the brain through several convergent pathways [22,105,106].

Thus, OMV exposure of the CNS is shaped not by a single microbial source but by anatomically distinct mucosal reservoirs that differ in proximity, trafficking routes, and vesicle composition. The oral cavity and olfactory–upper airway axis represent two particularly relevant niches, given their dense Gram-negative microbial communities and direct or indirect access to the brain via the trigeminal and olfactory nerves, vascular dissemination, and BBB transcytosis [73,107,108].

Multiple bacterial species residing within oral and olfactory niches are capable of releasing OMVs with the potential to influence host inflammation, metabolism, and proteostatic balance. Rather than implying causality for individual pathogens, these organisms collectively represent anatomically distributed reservoirs of vesicular signaling that may converge on vulnerable brain regions and contribute to cumulative vesicular load. Key examples of OMV-producing bacteria with reported links to AD-relevant neuroinflammatory, metabolic, or proteinopathic processes are summarized in Table 1.

### 4.1. Translocation Across Epithelial Barriers

OMVs must first cross epithelial interfaces at primary sites of microbial colonization, including nasal, oral, pulmonary, and gastrointestinal mucosa. Multiple lines of evidence show that vesicles penetrate these barriers through lipid raft-mediated endocytosis driven by vesicle enrichment in cholesterol and sphingolipids, through macropinocytosis and other clathrin-independent uptake routes, and through paracellular leakage that follows disruption of tight junctions [119,120,121]. Vesicle cargo, such as LPS, proteases, and adhesins, actively destabilizes tight junction proteins, including occludin and claudins, which increases mucosal permeability and facilitates further translocation [53,73,122]. Notably, this process is not acutely cytotoxic. Rather, it establishes a persistent low-grade barrier dysfunction that aligns with the chronic nature of AD [53,72].

After crossing the epithelium, vesicles access subepithelial immune and vascular compartments and can disseminate systemically [123,124]. In practical terms, different mucosal niches are likely to be dominated by distinct vesicle-producing communities. In the nasal cavity and upper airway, *Moraxella catarrhalis*, *Haemophilus influenzae*, and *Neisseria meningitidis* represent important sources of vesicles capable of engaging the olfactory neuroepithelium and nasopharyngeal mucosa [51,53,87,109,125]. Within the oral cavity, *Porphyromonas gingivalis* and other periodontal pathobionts release vesicles that penetrate the gingival vasculature, contributing to low-grade systemic inflammation [126,127]. Chronic respiratory infection with *Chlamydia pneumoniae* adds further complexity, as vesicle-like structures and infected cell-derived particles can enter circulation and interact with vascular and glial compartments [128,129]. Collectively, these organisms illustrate how anatomically distinct microbial reservoirs supply vesicles that converge on CNS entry points [15,130].

In vitro studies using human respiratory epithelial cell models demonstrate that OMVs derived from non-typeable *Haemophilus influenzae* (NTHi) are internalized via caveolin-associated lipid raft domains, enabling efficient host–cell interaction. These vesicles (20–200 nm) carry multiple virulence-associated factors, including adhesin P5, IgA endopeptidase, serine proteases, and heme-utilization proteins, and induce epithelial immune responses such as IL-8 secretion and LL-37 production [131].

Complementing these mechanistic findings, preclinical in vivo studies in murine models demonstrate that inhaled NTHi OMVs can actively drive airway inflammation. Aerosolized OMVs administered to sensitized mice induce airway hyper-responsiveness and neutrophilic inflammation, with transcriptional enrichment of IL-1β and IL-17 signaling pathways in lung tissue [125].

### 4.2. Vascular Dissemination and BBB Interaction

Once in circulation, OMVs engage vascular endothelial cells, including those that form the BBB. Vesicles activate the endothelium through TLR4 and NOD dependent signaling [53,126]. In a primary human BBB co-culture, *Porphyromonas gingivalis* vesicles or LPS reduced barrier integrity as shown by decreased trans-endothelial electrical resistance and re-localization of ZO-1, indicating tight-junction compromise in human brain microvascular endothelium [54,132]. In vivo, OMVs have been detected within brain regions following peripheral exposure and are associated with BBB disruption and neuroinflammatory responses, supporting their capacity to access and modulate CNS environments [17]. Consequences of this include increased expression of ICAM 1 and VCAM 1, elevated production of nitric oxide and ROS, disruption of tight junction complexes, and greater endothelial permeability [133,134,135]. In addition to promoting paracellular leakage, vesicles undergo active transcytosis across the BBB, a process enabled by their lipid-rich membranes and nanoscale dimensions. This mechanism is particularly relevant in the aging brain, where barrier integrity is already compromised by vascular dysfunction, cerebral hypoperfusion, and chronic inflammation. These features are common in prodromal and early AD and are expected to amplify vesicle entry into the CNS [15,136].

### 4.3. Role of OECs in Vesicle Uptake and Relay

Within the olfactory system, OECs occupy a highly active immunological and metabolic position at the interface between the peripheral and CNS. Rather than acting as passive barriers, this phagocytic glia internalizes OMVs and initiate inflammatory and metabolic signaling in response to vesicle exposure [137]. This protective role also makes them susceptible to intracellular accumulation of OMVs. Upon uptake, OECs show inflammatory activation, oxidative stress, and metabolic suppression, phenotypes that mirror changes observed in astrocytes and microglia during AD progression [53,137,138]. In this context, OECs may act not only as barriers but also as amplification hubs. They can internalize vesicles from the nasal compartment and relay inflammatory and metabolic stress signals into the olfactory bulb and downstream limbic structures [46,139,140]. This mechanism provides a biologically coherent link between peripheral infection, early olfactory dysfunction, and subsequent central neurodegeneration. The entry of vesicles into the olfactory bulb places microbial cargo in immediate proximity to limbic-connected circuits, consistent with the early network vulnerability described across olfactory, entorhinal, and hippocampal pathways [35,40,125].

Pathogen-specific distinctions are reflected in evidence describing vesicle-mediated epithelial entry, vascular interactions, glial responses, and proteinopathy-associated outcomes across major airway and oral pathobionts (Table 2).

## 5. CNS Glial Cells as Targets of OMVs in Neurodegeneration

Within the CNS, microglia and astrocytes constitute principal innate immune and metabolic regulatory cell populations. These glial cells are now recognized as active drivers of AD pathogenesis rather than passive responders to neuronal injury [8,10]. Once OMVs gain access to the brain through vascular or olfactory routes, they encounter glial cells as their first and most responsive targets. Owing to their high endocytic capacity, dense expression of pattern recognition receptors, and intimate contact with neurons and synapses, glia are uniquely susceptible to vesicle-mediated reprogramming [148,149]. Unlike transient exposure to soluble bacterial products, vesicle internalization establishes sustained intracellular immune activation and metabolic stress, driving long-lived pathological glial phenotypes that closely resemble those observed in an AD brain [14,17,53].

### 5.1. OMV-Induced Microglial Reprogramming: From Surveillance to Disease-Associated Phenotypes

Microglia are exquisitely sensitive to vesicle cargo and respond through multiple receptor pathways, including Toll-like receptors, NOD-like receptors, and inflammasome-associated sensors [150,151]. Vesicle internalization rapidly induces the expression of pro-inflammatory mediators such as TNF-α, IL-1β, IL-6, and type I interferons. These expressions promote transition from homeostatic surveillance states toward activated disease-associated phenotypes [53,152,153], with a defining feature of vesicle-stimulated microglia being the concurrent induction of lipid metabolic dysregulation [150]. Exposure to this environment suppresses oxidative phosphorylation and mitochondrial respiration, enhances glycolytic flux, impairs cholesterol efflux and fatty acid oxidation, and drives the accumulation of intracellular lipid droplets [43,64]. These changes mirror lipid droplet–accumulating microglial states described in aging and AD, which exhibit reduced phagocytic efficiency, impaired synaptic pruning, and exaggerated inflammatory signaling [146]. Importantly, vesicle-driven microglial activation is not self-limiting; instead, it establishes feed-forward inflammatory loops through continuous release of cytokines, ROS, nitric oxide, and bioactive lipid mediators that propagate neurotoxicity across local networks [152,153].

In middle-aged mice, oral administration of *Porphyromonas gingivalis* OMVs (4 mg/kg on alternate days for eight weeks) impaired spatial learning and memory and reduced hippocampal tight-junction gene expression, while elevating hippocampal IL-1β and NLRP3 signaling, consistent with vesicle-driven neuroinflammation in vivo. Conditioned media from OMV-stimulated microglia increased tau phosphorylation at Thr231 in neuronal cells, an effect attenuated by NLRP3 inhibition, thereby linking vesicle-primed microglial activation to tau-directed kinase signaling [17].

### 5.2. OMV-Triggered Astrocytic Reprogramming and the Decline of Neuronal Support Functions

Astrocytes play a central role in homeostatic functions. Such functions include metabolic coupling between vasculature and neurons, regulation of extracellular ion homeostasis, maintenance of neurotransmitter clearance, and provision of essential trophic support [154]. Exposure to vesicles profoundly disrupts the homeostatic functions of astrocytes, as they undergo a reactive transformation following their internalization. This transformation is characterized by suppression of mitochondrial respiration, enhanced reliance on glycolysis, reduction in lactate export to neurons, impaired lipid synthesis and trafficking, and downregulation of neurotrophic factor production [43,65,154]. These changes compromise the astrocyte–neuron metabolic shuttle, depriving neurons of essential energetic substrates and amplifying oxidative stress [155]. In parallel, vesicle-stimulated astrocytes secrete pro-inflammatory cytokines, chemokines, and lipid-derived mediators that reinforce microglial activation and destabilize synaptic microenvironments. Emerging evidence also indicates vesicles modulate astrocytic glutamate handling and potassium buffering, exacerbating excitotoxic stress at vulnerable synapses. Collectively, these effects convert astrocytes from metabolic protectors into active contributors to neurodegenerative cascades [65,156,157].

### 5.3. Convergence of Inflammation and Metabolism as a Central Pathological Axis

A defining feature of vesicle and glia interactions is the tight coupling of inflammatory signaling through metabolic reprogramming. Activation of NFκB, MAPK, and interferon pathways simultaneously suppresses mitochondrial oxidative metabolism and promote a glycolytic shift in both microglia and astrocytes [65,158]. This coordinated reprogramming favors chronic inflammatory states at the expense of reparative and neuroprotective functions. Under conditions of cerebral hypoperfusion and age-related vascular decline, which are common in prodromal AD, vesicle-driven metabolic stress is further amplified [159,160]. Hypoxia stabilizes HIF1α, reinforcing glycolytic dominance, impairing lipid oxidation, and enhancing inflammatory gene expression. In this context, vesicles act as powerful synergistic amplifiers of neuroimmune and metabolic failures, which signify early disease [158,161].

### 5.4. Apolipoprotein E Genotype as a Modifier of Vesicle-Induced Lipid Stress

**Figure 3:**
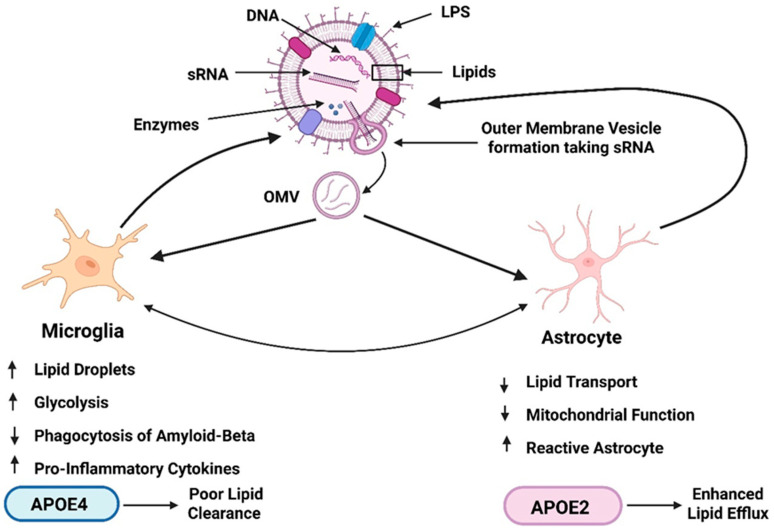
Vesicle-induced glial metabolic and lipid reprogramming modulated by *apolipoprotein E* genotype. Schematic illustrating how bacterial OMVs, carrying complex cargo including lipids, LPS, enzymes, bacterial DNA, and small RNAs, interact with CNS glial cells to drive metabolic and inflammatory reprogramming. Following entry into the brain, OMVs are preferentially internalized by microglia and astrocytes, triggering cell-type–specific responses. In microglia, vesicle exposure promotes lipid droplet accumulation, a shift toward glycolytic metabolism, increased pro-inflammatory cytokine production, and reduced phagocytic clearance of Aβ. In astrocytes, OMVs impair lipid transport and mitochondrial function while inducing reactive astrogliosis, thereby compromising metabolic support to neurons. These vesicle-induced effects are further shaped by *apolipoprotein E* genotype, with APOE4 associated with impaired lipid clearance and exacerbated lipid accumulation, and APOE2 linked to enhanced lipid efflux and relative metabolic resilience. Together, the figure illustrates how OMVs act as lipid-rich pathogenic units that couple infection-derived signals to glial dysfunction and genotype-dependent vulnerability in AD. Created in BioRender. Mathiske, T. (2026) https://BioRender.com/iail9tw.

Apolipoprotein E (APOE) genotype is the strongest genetic risk factor for late-onset AD and plays a central role in lipid transport, cholesterol homeostasis, and glial metabolic regulation [162]. These functions position APOE as a critical modifier within a vesicle-centric framework, particularly given the lipid-rich composition of bacterial OMVs. Rather than acting as an independent pathogenic driver, the APOE genotype is proposed to shape the cellular response to cumulative vesicular exposure by modulating lipid handling capacity, inflammatory tone, and metabolic resilience [163,164] (Figure 3).

In microglia, APOE4 expression is associated with impaired cholesterol efflux, reduced lipid turnover, and exaggerated accumulation of intracellular lipid droplets [94,165,166]. Within the context of OMV exposure, these deficits are predicted to exacerbate vesicle-induced lipid overload, reinforcing the transition toward disease-associated microglial phenotypes characterized by impaired phagocytosis, heightened inflammatory signaling, and reduced Aβ clearance. By contrast, APOE2-associated enhancement of lipid efflux and metabolic flexibility may partially buffer vesicle-driven lipid stress, attenuating inflammatory amplification and delaying downstream proteinopathy [165,167].

Astrocytic APOE-dependent effects further amplify these genotype-specific responses. Astrocytes are the primary source of APOE within the CNS and play a key role in distributing lipids to neurons [168,169,170,171]. Vesicle-induced disruption of astrocytic lipid trafficking and mitochondrial function is therefore predicted to have more severe consequences in APOE4 carriers, in whom lipid redistribution and metabolic support are already compromised. This convergence of vesicle-derived lipid burden and APOE4-associated transport inefficiency provides a mechanistic explanation for genotype-dependent vulnerability to early metabolic failure [172,173].

Importantly, within the context of vesicle exposure, APOE genotype does not initiate pathology but instead modifies the threshold and trajectory of vesicle-induced cellular stress. This framing reconciles genetic susceptibility with environmental and microbial contributions, offering a coherent explanation for inter-individual variability in disease onset, regional vulnerability, and response to intervention. It also provides a rationale for why lipid-targeted or vesicle-modulating therapies may exhibit differential efficacy across APOE genotypes [174,175,176,177].

### 5.5. Secondary Neurotoxicity and Network Propagation

The consequences of vesicle-induced glial reprogramming extend beyond local immune activation. Activated microglia and reactive astrocytes collaboratively shape a toxic extracellular milieu enriched in cytokines, oxidative mediators, aberrant lipid species, and misregulated neurotransmitters [159]. These factors collectively drive synaptic stripping, loss of dendritic spines, impaired long-term potentiation, mitochondrial dysfunction in adjacent neurons, and heightened susceptibility to Aβ and tau toxicity. Moreover, the release of glial-derived extracellular vesicles and inflammatory mediators enables pathology to propagate trans-synaptically and across anatomically connected networks, accelerating the stereotypical spatial progression of AD [178,179,180].

## 6. OMVs at the Convergence of Inflammation, Metabolism, and Protein Misfolding in AD

While neuroinflammation and metabolic dysfunction represent critical upstream drivers of AD, the irreversible clinical trajectory of the disorder is ultimately defined by the accumulation and spread of misfolded Aβ and hyperphosphorylated tau. A growing body of evidence indicates that OMVs are not merely indirect modulators of these proteinopathies through glial activation, but that they directly influence molecular machinery governing amyloidogenic processing, tau phosphorylation, and toxic aggregate propagation [17,181,182,183].

OMVs exert these effects through three tightly integrated mechanisms: (1) direct activation of amyloidogenic enzyme systems, (2) metabolic rewiring of lipid rafts and membrane microdomains that control amyloid precursor protein processing, and (3) inflammatory kinase signaling that accelerates tau hyperphosphorylation and misfolding. Together, these pathways position vesicles as systems-level accelerators of both major hallmarks of AD [184,185].

### 6.1. OMVs and Amyloidogenic Processing of APP

Aβ generation is governed by coordinated activity of β- and γ-secretases within cholesterol- and sphingolipid-enriched membrane microdomains known as lipid rafts [159]. Vesicles are highly enriched in bacterial lipids, LPS, and outer membrane phospholipids, making them potent modulators of host membrane architecture following cellular uptake [160]. Once internalized by astrocytes, microglia, or neurons, OMV-derived lipids and membrane components can incorporate into host plasma membranes, where they disrupt membrane organization and promote the clustering of lipid rafts. This reorganization enhances the spatial proximity of amyloid precursor protein with β-secretase within these microdomains, a configuration known to favor amyloidogenic processing [186,187]. In parallel, vesicle-associated LPS activates TLR-4 and NF-κB signaling, which transcriptionally upregulates β-secretase expression. These combined biophysical and transcriptional effects of vesicles, therefore, strongly bias amyloid precursor protein processing toward the amyloidogenic pathway [188].

Beyond enzymatic activation, vesicles impair Aβ clearance mechanisms. Microglial lipid droplet accumulation and mitochondrial suppression reduce phagocytic efficiency, while astrocytic metabolic reprogramming diminishes amyloid export and degradation capacity. These deficits decisively shift the balance of Aβ homeostasis toward extracellular accumulation and plaque nucleation [65,189,190].

### 6.2. OMVs as Platforms for Aβ Nucleation and Aggregation

Emerging biophysical evidence suggests microbial surfaces and vesicular membranes can act as heterogeneous nucleation platforms for Aβ aggregation [191]. Vesicles provide an ideal scaffold for this process due to their nanoscale size, high lipid content, and repetitive surface charge architecture [192,193]. Aβ peptides interacting with vesicle or membrane surfaces show markedly accelerated fibrillization, driven by membrane-assisted nucleation processes that stabilize oligomeric seeds and promote β-sheet ordering [194]. In this context, vesicles are not merely upstream inducers of Aβ production but may directly catalyze the earliest stages of Aβ aggregation. This offers a mechanistically coherent explanation for the frequent co-localization of Aβ deposition with regions of intense innate immune activation and microbial signal exposure [195,196].

### 6.3. OMV-Driven Kinase Activation and Tau-Hyperphosphorylation

Tau pathology is regulated by the balance between multiple serine/threonine kinases and phosphatases, many of which are sensitive to inflammatory and metabolic cues [197]. Through cytokine-dependent and oxidative stress–dependent pathways, vesicle exposure robustly activates key tau kinases, including glycogen synthase kinase-3β, cyclin-dependent kinase 5, p38 MAPK, and JNK [198,199,200]. Concurrently, vesicle-induced mitochondrial stress—amplified by associated NAD^+^ depletion—creates a cellular environment that favors kinase-dominant signaling over phosphatase activity, thereby shifting tau toward a more hyperphosphorylated state [201]. These combined effects promote tau detachment from microtubules, cytoskeletal destabilization, and formation of insoluble neurofibrillary tangles. Importantly, tau hyperphosphorylation is not restricted to neurons directly exposed to vesicles. Glial-derived inflammatory mediators and oxidative metabolites also propagate kinase activation trans-synaptically, enabling vesicle-triggered tauopathy to extend across anatomically connected networks [178,202].

Beyond their pathogenic effects, OMVs are increasingly recognized as potential therapeutic platforms capable of modulating inflammatory and proteostatic pathways implicated in neurodegeneration. Engineered OMVs have been used as drug-delivery vectors and vaccine scaffolds, enabling targeted transport of bioactive molecules while eliciting controlled immune responses. Commensal-derived OMVs additionally demonstrate anti-inflammatory and homeostatic actions within host tissues, suggesting scope to counter chronic neuroinflammation associated with aggregation-prone disorders [20]. Complementary evidence in PD indicates that manipulating OMV-mediated signaling along the gut–brain axis may limit inflammation-driven propagation of protein misfolding, underscoring the prospective therapeutic value of OMVs in conditions marked by α-synuclein or tau pathology [19,20].

### 6.4. Bidirectional Coupling Between Proteinopathy and Glial Metabolic Failure

Once initiated, Aβ- and tau-driven pathology further amplifies the neuroimmune and metabolic disturbances originally triggered by vesicles. Aβ oligomers potentiate microglial activation and impair astrocytic mitochondrial respiration, while pathological tau disrupts axonal trafficking of mitochondria and metabolic enzymes, compounding energy failure and accelerating neurodegeneration [203,204,205]. This establishes a bidirectional feed-forward loop in which vesicles may initiate pathology, and protein aggregates subsequently magnify glial dysfunction and energetic collapse [203,205,206]. This coupling helps explain why early infectious or inflammatory insults can produce delayed but relentless neurodegenerative trajectories, even in the absence of ongoing high-level microbial exposure.

### 6.5. Spatial Propagation of OMV-Primed Proteinopathies

Following olfactory or vascular entry, bacterial OMVs may interact with CNS-facing epithelial and myeloid-derived cells, and their pro-inflammatory cargo has the potential to activate glial pathways once within the brain. Although region-specific OMV-induced glial priming has not yet been directly demonstrated in human AD tissue, the anatomical overlap between early AD-affected circuits and proposed vesicle entry routes provides a biologically plausible framework [207,208]. Within this context, vesicle-driven inflammation and metabolic stress are hypothesized to establish permissive conditions for protein aggregate emergence and network propagation, rather than acting as independently sufficient triggers. This distinction emphasizes that the model proposed here remains mechanistically grounded but requires direct experimental validation in human systems [57,209,210,211].

### 6.6. Nucleic Acid Cargo of OMVs as Direct Drivers of Neuroinflammation

In addition to proteins and lipids, OMVs transport bacterial DNA fragments and small regulatory RNAs that act as potent innate immune stimuli [212]. Following vesicle internalization, these nucleic acids access endosomal and cytosolic pattern-recognition receptors, including TLR7, TLR8, RIG-I, and the cGAS–STING pathway. Activation of these sensors induces type I interferon responses and sustained inflammatory gene expression [66,213]. Chronic STING (Stimulator of Interferon Genes) activation has emerged as a key feature of age-related neuroinflammation and is increasingly implicated in neurodegenerative disease [214,215]. OMV-mediated delivery of bacterial DNA provides a plausible mechanistic bridge linking peripheral infection to the persistent interferon-stimulated gene signatures observed in AD transcriptomic datasets, given that OMVs carry bacterial DNA and potently activate PRR-dependent inflammatory pathways [53,135,187]. Importantly, nucleic acid-driven inflammation is resistant to resolution, further reinforcing chronic tendencies of vesicle-induced glial activation [209,216,217].

## 7. The Accumulative Vesicle Load Hypothesis

AD has traditionally been conceptualized through compartmentalized frameworks that emphasize individual pathological domains such as Aβ accumulation, tau hyperphosphorylation, neuroinflammation, or metabolic dysfunction in isolation [218,219]. However, these models struggle to account for the decades-long prodromal phase, the early vulnerability of olfactory and limbic circuits, and the profound coupling between infection, immunity, metabolism, and proteinopathy observed in the aging brain. An integrated system-level perspective is therefore required.

**Figure 4:**
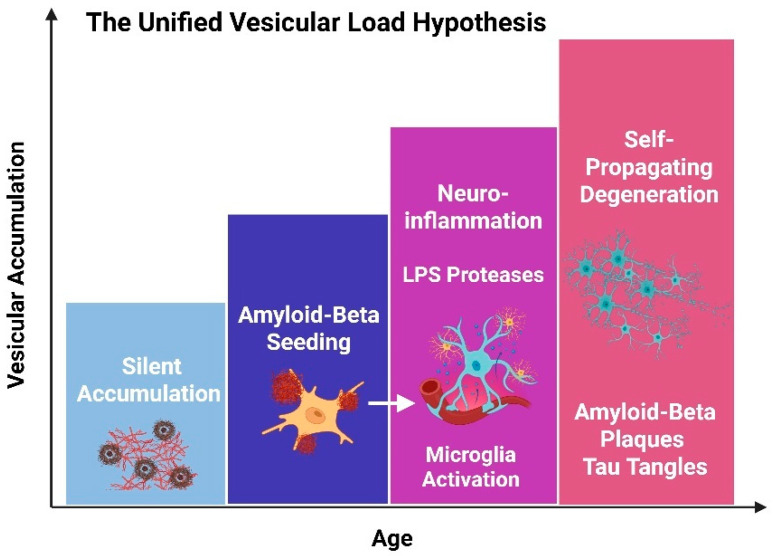
The Accumulative Vesicle Load Hypothesis. This schematic illustrates a proposed temporal model in which increasing vesicular pathogenic burden—including extracellular vesicles and microbe-derived vesicles—drives the progression of Alzheimer-related pathology across the lifespan. The *X*-axis (Age) represents the gradual advancement from early, clinically silent stages to late-stage neurodegeneration. The *Y*-axis (Vesicular Accumulation) reflects the progressive buildup of pathogenic vesicles, their inflammatory cargo, and their downstream effects on CNS cells. The first stage, Silent Accumulation, depicts low-level vesicular exposure that initiates subtle molecular changes without overt symptoms. As vesicular load increases, the system transitions to Aβ Seeding, where vesicle-associated Aβ and lipid cargo facilitate initial misfolding and deposition processes. Continued pathogenic vesicle exposure promotes Neuroinflammation, driven by microglial activation, LPS-containing vesicles, and vesicular proteases that amplify cytokine production and innate immune responses. In the final stage, Self-Propagating Degeneration, a high vesicular burden accelerates the spread of Aβ plaques, tau tangles, and other toxic aggregates, promoting a chronic degenerative cycle that is independent of the initial trigger. Together, these four stages illustrate how age-dependent vesicular accumulation may act as the unifying upstream force that links early silent pathology to late-stage neurodegeneration, thereby framing AD as a progressive disorder of vesicle-driven inflammatory and proteopathic stress. Created in BioRender. Mathiske, T. (2026) https://BioRender.com/iail9tw.

We therefore propose the Accumulative Vesicle Load Hypothesis, which postulates that bacterial OMVs act as systems-level integrators, unifying traditionally discrete pathogenic cascades into a coherent disease continuum [220,221,222]. In this model, vesicles act not merely as inflammatory triggers but as mobile, self-contained pathogenic units that physically transport, protect, and deliver complex microbial signals across peripheral–central boundaries [223]. Their nanoscale dimensions, lipid-rich composition, and resistance to enzymatic degradation enable persistent dissemination from mucosal and systemic infection reservoirs to vulnerable CNS entry points. Once within the brain, vesicles may initiate a cascade of tightly coupled cellular and molecular events that unfold across multiple hierarchical levels of neural organization [19,222,224]. The systems-level trajectory proposed by the Accumulative Vesicle Load Hypothesis is summarized schematically in Figure 4.

Much of the mechanistic understanding of this vesicle-driven neuroimmune and metabolic reprogramming originates from in vitro studies and animal experiments. Emerging associative evidence in humans, however, is in alignment with this model. Distinctive microbial compositions in nasal, oral, and airway regions, early olfactory impairment, endothelial activation, and transcriptomic signatures indicating glial inflammation and metabolic stress have been observed in individuals either at risk for or in the earliest stages of AD [47,225]. Although these observations do not establish causality, they are consistent with the preclinical data suggesting that ongoing, low-grade exposure to microbial vesicles may act as an upstream modulatory influence to shape regional vulnerability and influence disease trajectory well before overt cognitive symptoms [14,226,227].

The following phased description operationalizes the Accumulative Vesicle Load Hypothesis across peripheral, vascular, and CNS compartments.

### 7.1. Phase I: Peripheral Origin and Silent CNS Seeding

The earliest phase of AD is defined by chronic, often clinically silent, microbial activity at peripheral mucosal sites such as the nasal cavity, oral cavity, and gastrointestinal tract [74,193]. Sustained vesicle shedding establishes an ongoing flux of microbial signals into subepithelial tissues, vascular compartments, and the olfactory neuroepithelium. At this stage, CNS exposure to vesicles is low-level but persistent, enabling gradual glial priming without overt neurodegeneration [24]. Vesicle entry into the brain is via BBB transcytosis and, critically, the olfactory nerve–olfactory bulb axis. This allows concentrated microbial cargo to accumulate within the earliest AD-vulnerable networks in a process that establishes molecular seeding, whereby OECs, astrocytes and microglia and undergo reprogramming years before clinical symptoms emerge. The long latency between vesicle exposure and cognitive decline is proposed to reflect a prolonged period of subthreshold pathological conditioning rather than an absence of disease activity [135,228,229].

### 7.2. Phase II: Glial Reprogramming and Metabolic Collapse

With continued vesicle exposure, glial cells undergo progressive and self-reinforcing phenotypic conversion. Microglia transition from homeostatic surveillance states to inflammatory, lipid-laden disease-associated phenotypes with compromised phagocytic capacity and heightened cytokine output. Astrocytes, in parallel, lose their metabolic and neurotrophic support functions as mitochondrial respiration declines and glycolytic dependence increases [60,189,230]. This phase is marked by the collapse of neuroimmune–metabolic homeostasis, characterized by persistent activation of innate immune signaling pathways, suppression of oxidative phosphorylation, disruption of lipid handling, and failure of astrocyte–neuron metabolic coupling [231,232,233]. Age-related vascular insufficiency and cerebral hypoperfusion further potentiate this metabolic collapse by amplifying hypoxia-driven reprogramming. At this stage, neural networks remain largely structurally intact but become energetically fragile and highly sensitive to secondary insults [206,228,234].

### 7.3. Phase III: Proteinopathy Initiation and Network Destabilization

As neuroimmune–metabolic failure intensifies, vesicle-driven molecular perturbations converge on the core Aβ and tau proteinopathies of AD. Enhanced amyloidogenic processing of amyloid precursor protein, impaired Aβ clearance, and vesicle-facilitated Aβ nucleation collectively promote the emergence of extracellular plaques [235,236]. Simultaneously, inflammatory kinase activation and phosphatase suppression accelerate tau hyperphosphorylation, cytoskeletal destabilization, and intraneuronal tangle formation. Once initiated, these protein aggregates acquire partial biological autonomy, enabling their trans-synaptic spread across connected neural networks in a prion-like manner [206,234,237,238]. Crucially, their initial emergence is conditioned by permissive environments created through vesicle-driven glial and metabolic dysfunction. At the network level, this phase corresponds to the earliest detectable synaptic failure, impaired plasticity, and disconnection of limbic and associative cortical circuits. Clinically, this aligns with the transition from prodromal to mild cognitive impairment [234,237,239].

### 7.4. Phase IV: Self-Propagating Degeneration and Clinical Dementia

In the final phase, bidirectional amplification loops between proteinopathy, glial dysfunction, and neuronal metabolic failure become self-sustaining. Vesicle exposure may continue to contribute to this cycle, but disease progression is no longer contingent upon ongoing high-level microbial input [60,206,228]. At this stage, neurodegeneration becomes spatially widespread and clinically manifests as progressive dementia. Importantly, this model explains why antimicrobial or anti-inflammatory interventions initiated late in the disease course often show limited efficacy: by this point, the pathological system has transitioned from vesicle-dependent initiation to aggregate-driven autonomous degeneration [234].

### 7.5. Systems-Level Implications of the OMV Model

The Accumulative Vesicle Load Hypothesis presented here proposes a reframing of AD as a systems-level failure of neuroimmune–metabolic regulation, potentially initiated or conditioned by infectious vesicular inputs. It offers a unifying conceptual structure that links multiple, traditionally competing, hypotheses by positioning them as interdependent components of a single pathological cascade [240,241]. Within this framework, the infectious hypothesis is supported by vesicle-mediated delivery of microbial signals; the neuroinflammatory hypothesis is reflected in sustained glial reprogramming; the metabolic hypothesis is embedded in vesicle-associated mitochondrial and lipid dysfunction; and the Aβ and tau hypotheses are placed downstream as execution phases rather than primary initiating events [43]. Importantly, this model provides a mechanistic rationale for pronounced anatomical selectivity of early Alzheimer’s pathology within olfactory–entorhinal–hippocampal circuits, which occupy privileged positions along proposed vesicle entry and propagation pathways [23,24,46,210].

### 7.6. Translating the Integrated Model to Testable Predictions

The Accumulative Vesicle Load Hypothesis generates a set of explicit and falsifiable predictions that specify how vesicle signatures, glial responses, and network vulnerability should align if vesicle-driven conditioning precedes classical Alzheimer pathology. These predictions define concrete points at which the model can be supported or refuted across molecular, anatomical, and clinical domains [23,43,46,205]. Table 3 summarizes the core predictions generated by the Accumulative Vesicle Load Hypothesis and outlines the specific molecular, glial, and network-level phenomena that should be observable if the model is correct.

Although direct quantification of bacterial OMVs in early human AD brain tissue remains limited, convergent evidence from in vitro models, animal studies, vascular biology, and human transcriptomic datasets supports the biological plausibility of vesicle-mediated conditioning. The model therefore serves as a mechanistically grounded and testable framework rather than a claim of established causality.

## 8. From Mechanism to Medicine: A Mechanistic Rationale for OMVs as Biomarker Candidates

An ideal biomarker for AD must satisfy several stringent criteria: it should reflect upstream disease mechanisms, be detectable during the prodromal phase, be minimally invasive to access, and provide dynamic information about disease trajectory and treatment response [244,245,246]. OMVs uniquely fulfill all these requirements. Unlike static host-derived biomarkers that often reflect downstream neurodegeneration, vesicles constitute mobile, mechanistically active disease drivers that integrate microbial burden, immune activation, and metabolic stress into a single measurable entity [135,247].

As mentioned, vesicle size, lipid encapsulation, and cargo stability allow them to persist within biological fluids. Thus, vesicles are accessible in nasal secretions, saliva, blood, and cerebrospinal fluid. Vesicles also retain molecular fingerprints of their bacterial origin, including LPS structure, outer membrane proteins, and nucleic acids, while simultaneously incorporating host-derived inflammatory and metabolic signatures following cellular uptake. This dual microbial–host identity positions them as composite biomarkers capable of reporting both infectious burden and CNS biological response [135,247]. In the context of AD, vesicle signatures will likely reflect contributions from specific microbial reservoirs. Nasal and olfactory vesicles are expected to be enriched for material derived from upper airway pathobionts such as *Moraxella catarrhalis*, *Haemophilus influenzae*, and *Neisseria meningitidis*. This would provide direct evidentiary links between changes in the nasal microbiota and early olfactory and limbic network vulnerability [109,110,125,131,247,248,249]. By contrast, blood-derived vesicles may carry a mixed signature incorporating periodontal organisms such as *Porphyromonas gingivalis* together with vesicle-like particles arising during chronic *Chlamydia pneumoniae* infection. Recognizing these pathogen-specific patterns is important for interpreting vesicle-based biomarkers and distinguishing airway- and oral-driven signals along the nasal–brain and oral–systemic–brain axes [27,74].

### 8.1. Nasal and Olfactory OMVs as Ultra-Early Biomarkers

Nasal OMVs represent a uniquely valuable biomarker modality within the Accumulative Vesicle Load Hypothesis because they provide the most direct and accessible readout of vesicular activity at the host–microbe interface that lies upstream of widespread neurodegenerative change [250]. The nasal compartment captures vesicle populations close to their point of origin and to their earliest routes of central access, allowing nasal OMVs profiling to identify pathogenic vesicular pressure long before proteinopathy or irreversible neuronal loss becomes apparent. Unlike blood or CSF, which integrate signals from multiple anatomical reservoirs, nasal samples offer a high-resolution window into upper airway microbial dynamics and their immediate consequences for local inflammatory and metabolic stress [251,252].

A central advantage of nasal OMVs lies in their capacity to encapsulate both microbial and host-derived signatures. Their lipid and protein composition, together with bacterial nucleic acids and virulence factors, preserve information about airway pathobionts and their vesicle shedding behavior. At the same time, the host components incorporated into these vesicles, including cytokine-associated proteins, complement factors, reactive lipids, and regulatory RNAs, provide a molecular readout of epithelial and glial-like cellular responses. When analyzed through multi-omics platforms, nasal OMVs therefore offer an integrated molecular fingerprint that reflects the combined effects of microbial burden, innate immune activation, and early metabolic reprogramming. Functional assays that quantify TLR2, TLR4, or cGAS–STING activation could further extend this approach by capturing the inflammatory potency of vesicle cargo rather than its abundance alone [221,250,252].

Quantitative evidence supports the feasibility of nasal or upper-airway vesicle profiling. In human nasopharyngeal epithelial systems, non-typeable *Haemophilus influenzae* OMVs measuring 20–200 nm bind and are internalized via caveolin-rich lipid rafts, eliciting significant IL-8 release and induction of LL-37, while carrying defined virulence cargo including adhesin P5, IgA endopeptidase, a serine protease, and a heme-utilization protein. In vivo, aerosolized NTHi OMVs delivered to sensitized mice provoke robust IL-1β and IL-17 pathway activation and neutrophilic inflammation in lung tissue, demonstrating that airway vesicles generate measurable mucosal immune signatures that are biologically detectable at the epithelial interface. These findings establish that nasal sampling can capture stable vesicle populations with quantifiable microbial and host-response markers, supporting their use as ultra-early readouts within vesicle-centric AD frameworks [125,131,253].

The clinical utility of nasal OMVs emerges from their accessibility, stability, and suitability for longitudinal monitoring. Nasal sampling is rapid and minimally invasive, enabling repeated collection across preclinical, prodromal, and early symptomatic stages. This facilitates temporal tracking of vesicle burden, cargo composition, and inflammatory bioactivity, allowing researchers to detect accelerating patterns of vesicular activity that may precede cognitive decline [254]. In individuals with mild cognitive impairment, nasal OMVs may help to differentiate vesicle-high and vesicle-low endotypes, thereby supporting precision stratification of patients according to their neuroimmune-metabolic vulnerability. When combined with quantitative smell testing, blood-based biomarkers, and neuroimaging, nasal vesicle signatures can contribute to layered risk-prediction frameworks grounded in mechanistic biology [254].

The interpretation of nasal vesicle activity depends on its temporal trajectory and its relationship with other fluid biomarkers. A persistently elevated nasal vesicle signature in cognitively intact individuals suggests early vesicle exposure and glial priming consistent with the earliest phase of the Accumulative Vesicle Load Hypothesis. A pattern in which nasal vesicle activity increases before detectable changes in blood or CSF would support the proposed upstream localization of airway-derived vesicle burden [250]. Conversely, discordant findings such as low nasal vesicle activity in the presence of a high plasma vesicle load may indicate that oral or systemic reservoirs are contributing more strongly to the cumulative vesicular burden, or that pre-analytical variability has influenced the measurement and needs to be reviewed. For this reason, it is essential to maintain careful control of sampling conditions, storage parameters, and common inflammatory confounders, including acute rhinitis and environmental exposures [250]. Taken together, nasal OMVs represent a mechanistically grounded and clinically scalable biomarker class capable of capturing the earliest vesicular pressures acting on vulnerable neural networks. By providing an integrated readout of microbial inputs and host responses at a critical neuroimmune interface, they offer a promising platform for ultra-early detection, precision stratification, and pharmacodynamic monitoring within the broader vesicle-centric framework of AD pathogenesis [250].

Saliva also represents a valuable and easily accessible biofluid for vesicle-based assessment. It can be collected repeatedly with minimal burden, and it may capture vesicles released from periodontal and oral reservoirs that contribute to systemic inflammatory tone. Within the Accumulative Vesicle Load Hypothesis, saliva-derived vesicles are expected to complement nasal profiles by reflecting microbial and host signals arising from the oral systemic axis, especially in individuals with chronic periodontal disease or elevated oral vesicle activity.

These translational applications remain prospective and will require validation in longitudinal human cohorts to determine sensitivity, specificity, and predictive value relative to established AD biomarkers.

In addition to their diagnostic potential, OMVs are increasingly considered as therapeutic candidates in neurodegenerative disease. Recent advances in OMV engineering enable the loading of anti-inflammatory cargo, small molecules, or RNA therapeutics into vesicles designed to access CNS-adjacent tissues. Their nanoscale size, immunomodulatory capacity, and ability to cross epithelial and endothelial barriers position OMVs as promising delivery systems for agents targeting early neuroinflammation and protein aggregation. Further, beneficial OMVs produced by commensal microbiota demonstrate natural homeostatic effects on host immune tone, suggesting potential translational routes for OMV-based interventions aimed at reducing neuroinflammation or modulating misfolded-protein propagation [17].

### 8.2. Blood-Derived OMVs as Systemic Indicators of Neuroimmune–Metabolic Stress

Circulating vesicles provide a complementary systemic biomarker layer reflecting the dynamic interplay between peripheral infection, vascular inflammation, and CNS vulnerability. Blood-derived vesicles can be repeatedly sampled and quantified, offering a practical platform for longitudinal disease monitoring. In the context of the Accumulative Vesicle Load Hypothesis, plasma vesicles are predicted to correlate with endothelial activation, BBB permeability, systemic inflammatory tone, and glial priming states within the brain [255,256]. Importantly, blood vesicles may serve as bridging biomarkers that link peripheral infection reservoirs to central neurodegeneration, enabling earlier therapeutic intervention before irreversible neuronal damage occurs [255,257].

### 8.3. CSF OMVs as Direct Reporters of Central Vesicular Pathology

Vesicles detected in CSF represent the most direct window into intracranial vesicular activity. Unlike total CSF cytokines or soluble Aβ and tau levels, CSF-derived vesicles preserve information about cellular origin, membrane composition, and active cargo delivery mechanisms [255,256]. In this framework, CSF vesicle analysis may provide a means to distinguish infection-driven inflammatory Alzheimer’s subtypes from purely genetic or vascular forms. They also quantify ongoing vesicle-mediated neuroimmune and metabolic stress and provide early pharmacodynamic readouts for therapies targeting vesicle biogenesis, uptake, or glial reprogramming. When combined with established CSF Aβ and tau assays, vesicles could add a causal layer of biological interpretation to existing diagnostics [255,256].

### 8.4. Multi-Omics Profiling of OMVs for Precision Stratification

A particularly powerful translational application of vesicles lies in their compatibility with high-resolution multi-omics technologies. Vesicles can be subjected to proteomic, lipidomic, and transcriptomic profiling, enabling comprehensive molecular fingerprinting that captures both microbial virulence factors and host stress signatures [255,257]. Multi-omics vesicle profiling offers a unique opportunity to define molecular Alzheimer’s endotypes, moving beyond one-size-fits-all diagnostic models. Such stratification could explain heterogeneity in disease progression, therapeutic response, and vulnerability to metabolic or inflammatory stressors.

### 8.5. OMVs as Pharmacodynamic Biomarkers

Beyond early diagnosis, vesicles are ideally positioned as treatment-response biomarkers. Because they sit upstream of Aβ and tau accumulation, changes in vesicle load, cargo composition, or uptake kinetics could provide earlier indicators of therapeutic efficacy than traditional proteinopathy-based readouts [255,257]. Potential intervention-linked applications include monitoring response to antimicrobial or microbiome-modulating therapies, assessing efficacy of vesicle-targeted inhibitors, and tracking metabolic rescue strategies aimed at restoring glial bioenergetics. In this role, vesicles function not merely as passive disease reporters but as real-time biosensors of upstream pathogenic pressure [255].

### 8.6. Integration with Clinical Smell Testing and Digital Biomarkers

A key translational strength of the Accumulative Vesicle Load Hypothesis is its natural integration with olfactory testing and digital phenotyping. Quantitative smell function assessments can capture early network-level dysfunction within olfactory–limbic circuits, while nasal vesicle profiling provides a molecular correlate of this functional impairment. When combined with cognitive screening, imaging, and wearable digital biomarkers, vesicles could contribute to a multilayered early detection platform that is both mechanistically grounded and clinically scalable [57].

## 9. From Pathogenic Vesicles to Actionable Drug Targets

The therapeutic considerations outlined below represent conceptual extensions of the Accumulative Vesicle Load Hypothesis rather than established clinical strategies. At present, no approved AD therapies directly target bacterial vesicle biology. The following framework is intended to illustrate how vesicle-centered mechanisms may inform future interventional design, pending rigorous experimental and clinical validation.

A central strength of the Accumulative Vesicle Load Hypothesis is that it repositions AD as a disorder with multiple therapeutically accessible entry points upstream of irreversible neurodegenerationgies [258]. Therapeutic targeting of the vesicle axis can be conceptualized across four complementary levels: (1) suppression of vesicle biogenesis, (2) blockade of vesicle uptake and trafficking, (3) neutralization of vesicle cargo, and (4) rescue of glial metabolic and immune dysfunction [259]. It further has direct translational implications for AD, spanning early detection, patient stratification, and therapeutic intervention.

Table 4 summarizes key translational applications of OMVs in AD, highlighting their potential roles as biomarkers, pharmacodynamic readouts, and therapeutic targets. These applications are organized across the disease continuum, from preclinical risk identification and prodromal stratification to treatment monitoring and combination therapy design. Rather than representing isolated use cases, these translational avenues reflect different points of engagement with a common vesicle-driven pathogenic axis.

### 9.1. Inhibition of OMV Biogenesis at the Microbial Source

Vesicle production is an active, regulated process rather than a passive by-product of bacterial growth. Vesiculation is enhanced by envelope stress, alterations in membrane asymmetry, and disruption of peptidoglycan–outer membrane linkages [163,266]. Targeting these pathways offers the earliest disease-modifying strategy, as it reduces pathogenic vesicle flux before CNS exposure occurs. Potential approaches include sublethal antimicrobial modulation to suppress vesiculation without catastrophic microbiome disruption, inhibitors of bacterial envelope stress responses to attenuate hypervesiculation, and selective microbiome editing to reduce vesicle-producing pathobionts while preserving commensal species [22,193,217]. Within the Accumulative Vesicle Load Hypothesis, such strategies are hypothesized to be most effective during preclinical and prodromal stages, before proteinopathy becomes self-propagating [267].

### 9.2. Blockade of OMV Uptake and CNS Entry

As vesicles exploit host endocytic machinery and lipid-raft-dependent transport mechanisms, host-directed therapies aimed at preventing vesicle internalization and brain entry represent a second major therapeutic layer. Intervention points include disruption of lipid rafts to reduce cholesterol-dependent uptake, modulation of endocytic pathways to limit vesicle internalization by endothelial cells and glia, and stabilization of epithelial and BBB tight junctions to reduce paracellular leakage [57,119,135,268]. Targeting the olfactory route, including modulation of nasal epithelial permeability and OEC activation, is particularly relevant for early intervention because this interface represents one of the earliest and most direct points of contact between environmental signals and the CNS. The dynamic and accessible nature of the nasal epithelium, coupled with the gatekeeping role of OECs, provides an opportunity to intercept vesicle-mediated neuroimmune and metabolic perturbations before the establishment of widespread neurovascular dysfunction or irreversible neurodegeneration [221,258,269].

### 9.3. Neutralization of OMV Cargo

Even after CNS entry, the biological impact of vesicles remains dependent on the activity of their molecular cargo. Neutralizing immunogenic and metabolic toxicity of vesicle constituents, therefore, offers a third layer of therapeutic intervention [270,271]. This includes LPS and lipoprotein neutralization to reduce Toll-like receptor-driven glial activation, protease inhibition to limit extracellular matrix degradation and synaptic destabilization, scavenging of oxidized lipids and ceramides to mitigate lipid toxicity, and sequestration of bacterial nucleic acids to reduce intracellular innate immune activation. These approaches do not require eradication of the microbial source and may be particularly valuable in later disease stages where vesicle exposure continues, but proteinopathy has already been initiated [272,273,274].

### 9.4. Metabolic and Immune Rescue of OMV-Reprogrammed Glia

Given that vesicles may operate as system-level drivers to rewire both immune and metabolic pathways, glial rescue strategies represent a powerful downstream intervention point. These approaches aim to restore glial bioenergetics, suppress chronic inflammatory signaling, and re-establish neuroprotective support functions [275,276,277]. Therapeutic approaches include mitochondrial metabolic rescue to enhance oxidative phosphorylation and NAD^+^ availability, lipid flux normalization to restore astrocytic lipid synthesis and microglial lipid clearance, reversal of chronic microglial activation to shift disease-associated phenotypes back toward homeostatic states, and restoration of astrocyte–neuron metabolic coupling to normalize lactate and lipid shuttling [278,279]. Within the Accumulative Vesicle Load Hypothesis, these strategies are hypothesized to lower neuronal vulnerability to Aβ and tau toxicity, even when protein aggregates are already present [228,280,281,282,283,284].

### 9.5. Targeting OMVs in Combination with Anti-Aβ and Anti-Tau Therapies

Rather than displacing Aβ- and tau-targeted therapies, a vesicle-centered approach may be most impactful when integrated within combination treatment paradigms that address upstream inflammatory and metabolic conditioning alongside downstream protein aggregation. In this framework, vesicle modulation is conceptualized as a complementary strategy aimed at reducing the permissive biological environment in which classical proteinopathies emerge and propagate.

The vesicle model presented here does not displace proteinopathy-targeted therapies; it suggests redefining their optimal positioning within a combination treatment paradigm. By reducing vesicle-driven upstream pathogenic pressure, the threshold for therapeutic efficacy of anti-Aβ and anti-tau agents is likely to be substantially lowered [178,240]. This suggests a rational staged or combinatorial strategy: early suppression of vesicle production and trafficking, concurrent rescue of glial metabolic and immune dysfunction, and targeted clearance or neutralization of residual Aβ and tau aggregates. Such multi-axis intervention directly addresses the repeated failure of single-target therapies in AD by acknowledging the multi-layered, system-level nature of disease progression [240,285].

### 9.6. Nasal and Microbiome-Targeted Therapeutics

A uniquely actionable implication of the Accumulative Vesicle Load Hypothesis is the therapeutic relevance of the nasal and upper airway microbiome. Local interventions at this interface offer the possibility of directly interrupting early stages of vesicle-driven neuroinvasion [286]. Potential strategies include topical antimicrobial or anti-vesiculation agents, targeted probiotic or bacteriophage approaches to alter vesicle-producing microbial communities, and nasal barrier-strengthening formulations to limit vesicle translocation. These approaches are uniquely scalable and could be integrated with longitudinal nasal vesicle biomarker monitoring, enabling true precision intervention at the earliest detectable disease stages [287,288,289].

## 10. Conclusions

This review examines emerging evidence that microbial OMVs may contribute to system-level vulnerability in AD by linking peripheral microbial activity with central neuroimmune, metabolic, and proteostatic pathways. By integrating infection biology with glial reprogramming, metabolic failure, and downstream Aβ and tau pathology, the Accumulative Vesicle Load Hypothesis proposes a coherent, temporally ordered framework that may help explain the long prodromal period, early regional selectivity, and limited efficacy of late, single-target interventions. Within this model, vesicle-derived signatures in nasal–oral fluid, blood, and cerebrospinal fluid may provide insight into upstream disease processes, creating opportunities for earlier detection, molecular stratification, and pharmacodynamic monitoring pending longitudinal validation. Therapeutically, targeting vesicle biogenesis, CNS entry, cargo toxicity, or glial metabolic resilience represents conceptual intervention points upstream of irreversible proteinopathy. Key priorities for future work include longitudinal human studies linking vesicle signatures to disease trajectory, refined characterization of pathogen-specific vesicle cargo, and mechanistic analyses of glial responses at single-cell and single-vesicle resolution. Proof-of-concept interventions that modify vesicle exposure or uptake will be essential for determining whether modulation of this upstream axis can meaningfully influence downstream proteinopathy. Taken together, current evidence supports OMVs as biologically plausible contributors at the interface between microbial ecology and brain aging, providing a testable framework for mechanistic investigation and translational exploration. Although this model offers an integrative perspective, alternative interpretations remain possible, including those in which vesicles function primarily as modulators rather than initiators of early pathology. Future human studies will therefore be essential to clarify the relative contribution of vesicle exposure in comparison with genetic, vascular, and age-related factors.

## Figures and Tables

**Table 1:** OMV-producing bacteria from oral and olfactory niches linked to Alzheimer’s pathogenesis.

Bacterial Species	Primary Niche	Major OMV Cargo Features	CNS Access Route	Key AD-Relevant Effects
*M. catarrhalis* [109,110,111]	Upper airway, nasopharynx	LPS, adhesins, porins, proteases	Olfactory nerve pathway (bypassing the BBB); may contribute to secondary BBB disruption	Microglial activation, nasal inflammation, barrier disruption
*H. influenzae* [112]	Nasopharynx, airway	LOS, outer membrane proteins, proteases	Olfactory + vascular	Endothelial activation, microglial priming, neuroinflammation
*N. meningitidis* [113,114]	Nasopharynx, blood	LOS, RTX toxins, porins	BBB transcytosis	Tight junction breakdown, neuroinflammation
*P. gingivalis* [74,115]	Oral cavity, periodontium	Gingipains, LPS, fimbrial proteins	Oral → systemic → brain	Aβ promotion, tau phosphorylation, chronic inflammation
*C. pneumoniae* [116,117,118]	Lung, intracellular	Vesicle-like extracellular structures, LPS-like molecules	Vascular + glial infection and olfactory/trigeminal nerve	Astrocyte and microglial infection, Aβ accumulation

**Table 2:** Evidence across pathogens for vesicle-mediated entry and early neuroimmune interaction.

Pathogen	CNS Access/BBB Interaction	Glial Reprogramming	Human Tissue/Biofluid/Clinical	Overall Strength
*Porphyromonas gingivalis* (OMVs) [17,141]	BBB compromise in human BBB models; reduced TEER and ZO-1 resocialization	↑ tau Thr231 phosphorylation; ↑ Aβ production in models	Bacterial components/antigens reported in AD brains; association studies	Strongest overall mechanistic portfolio among pathogens reviewed
*Neisseria meningitidis* (OMVs) [113]	MMP-8-mediated occludin cleavage; ↑ permeability in human brain endothelial cells	Indirect, via inflammatory kinase pathways	Human meningitis literature supports BBB injury mechanisms	Strong BBB mechanistic support; limited direct AD linkage
*Haemophilus influenzae* (NTHi) (OMVs) [131,142]	Epithelial uptake and barrier signaling; inhalation model shows IL-1β/IL-17 axes	No direct Aβ/tau yet; downstream inflammatory potential	Airway biology; no direct AD clinical signal	Moderate airway evidence; AD link speculative
*Moraxella catarrhalis* (Whole bacterium) [84,143,144]	CEACAM-mediated adhesion; OMV virulence cargo	No direct Aβ/tau	Upper-airway colonizer; no AD clinical data	Emerging airway OMV biology; AD link currently weak
*Chlamydia* spp. (VLPs/host EVs) [145,146,147]	Olfactory and trigeminal nerve access shown in rodents	Induces Alzheimer-like Aβ plaques in mice	Reports of detection in human AD brains	Distinct intracellular biology; comparative relevance

**Table 3:** Testable predictions derived from the Accumulative Vesicle Load Hypothesis.

Prediction	Description
Early vesicle signatures precede overt proteinopathy	Vesicle-associated markers should be detectable in nasal fluid, blood, or CSF during the preclinical and prodromal phases, appearing before substantial Aβ or tau accumulation [242].
Olfactory network vulnerability correlates with vesicle exposure	Olfactory connected regions (piriform, entorhinal, hippocampal networks) should exhibit the earliest glial and metabolic abnormalities in individuals with high vesicle burden [243].
Glial metabolic reprogramming precedes irreversible neurodegeneration	Microglia and astrocytes should show stable inflammatory and metabolic shifts before any measurable neuronal loss; if these early changes are absent, the proposed sequence is challenged [9].
Interruption of vesicle biogenesis or uptake attenuates downstream pathology	Blocking vesicle production or limiting CNS entry should reduce glial activation, dampen metabolic dysfunction, and mitigate later Aβ and tau pathology [9].
Vesicle burden modifies disease trajectory rather than acting as a sole trigger	Vesicle signatures should interact with genetic, vascular, and aging factors to produce varied disease trajectories; uniform progression would argue against a vesicle modulated model [243].

**Table 4:** Translational applications of OMVs in AD.

Application	Biological Sample	OMV Feature Measured	Clinical Utility
Ultra-early screening [250]	Nasal fluid/Saliva	OMV concentration, LPS, miRNA	Preclinical AD risk detection
Disease monitoring [260,261]	Blood	Inflammatory OMV cargo	Longitudinal progression tracking
CNS pathology [262,263,264]	CSF	Glial-derived OMVs, tau/Aβ associated cargo	AD subtype stratification
Treatment response [111,113,265]	Blood, nasal fluid	OMV load reduction	Pharmacodynamic biomarker
Microbiome-targeted therapy [73]	Nasal/oral samples	Pathogen-specific OMVs	Precision interception

## Data Availability

No new data were created or analyzed in this study. Data sharing is not applicable to this article.

## References

[1] Iliyasu M.O., Musa S.A., Oladele S.B., Iliya A.I. (2023). Amyloid-beta aggregation implicates multiple pathways in Alzheimer’s disease: Understanding the mechanisms. Front. Neurosci..

[2] Anitha K., Singh M.K., Kohat K., Sri Varshini T., Chenchula S., Padmavathi R., Amerneni L.S., Vishnu Vardhan K., Mythili Bai K., Chavan M.R. (2025). Recent Insights into the Neurobiology of Alzheimer’s Disease and Advanced Treatment Strategies. Mol. Neurobiol..

[3] Bloom G.S. (2014). Amyloid-β and tau: The trigger and bullet in Alzheimer disease pathogenesis. JAMA Neurol..

[4] Chen Y., Jin H., Chen J., Li J., Găman M.A., Zou Z. (2025). The multifaceted roles of apolipoprotein E4 in Alzheimer’s disease pathology and potential therapeutic strategies. Cell Death Discov..

[5] Cornitius C.P., Perez E.K., Lee S.C. (2025). Microbial links to Alzheimer’s disease. PLoS Pathog..

[6] Zhang S., Gao Y., Zhao Y., Huang T.Y., Zheng Q., Wang X. (2025). Peripheral and central neuroimmune mechanisms in Alzheimer’s disease pathogenesis. Mol. Neurodegener..

[7] Seo D.O., Holtzman D.M. (2024). Current understanding of the Alzheimer’s disease-associated microbiome and therapeutic strategies. Exp. Mol. Med..

[8] Deng Q., Wu C., Parker E., Liu T.C., Duan R., Yang L. (2024). Microglia and Astrocytes in Alzheimer’s Disease: Significance and Summary of Recent Advances. Aging Dis..

[9] Gao R., Gao Y., Su W., Wang R. (2025). Decoding Microglial Polarization and Metabolic Reprogramming in Neurodegenerative Diseases: Implications for Disease Progression and Therapy. Aging Dis..

[10] Han J., Zhang Z., Zhang P., Yu Q., Cheng Q., Lu Z., Zong S. (2025). The roles of microglia and astrocytes in neuroinflammation of Alzheimer’s disease. Front. Neurosci..

[11] Monzón-Sandoval J., Burlacu E., Agarwal D., Handel A.E., Wei L., Davis J., Cowley S.A., Cader M.Z., Webber C. (2022). Lipopolysaccharide distinctively alters human microglia transcriptomes to resemble microglia from Alzheimer’s disease mouse models. Dis. Model. Mech..

[12] Xingi E., Koutsoudaki P.N., Thanou I., Phan M.S., Margariti M., Scheller A., Tinevez J.Y., Kirchhoff F., Thomaidou D. (2023). LPS-Induced Systemic Inflammation Affects the Dynamic Interactions of Astrocytes and Microglia with the Vasculature of the Mouse Brain Cortex. Cells.

[13] Loh J.S., Mak W.Q., Tan L.K.S., Ng C.X., Chan H.H., Yeow S.H., Foo J.B., Ong Y.S., How C.W., Khaw K.Y. (2024). Microbiota-gut-brain axis and its therapeutic applications in neurodegenerative diseases. Signal Transduct. Target. Ther..

[14] Butler C.A., Ciccotosto G.D., Rygh N., Bijlsma E., Dashper S.G., Brown A.C. (2024). Bacterial Membrane Vesicles: The Missing Link Between Bacterial Infection and Alzheimer Disease. J. Infect. Dis..

[15] Roier S., Leitner D.R., Iwashkiw J., Schild-Prüfert K., Feldman M.F., Krohne G., Reidl J., Schild S. (2012). Intranasal immunization with nontypeable Haemophilus influenzae outer membrane vesicles induces cross-protective immunity in mice. PLoS ONE.

[16] Wei S., Ma X., Chen Y., Wang J., Hu L., Liu Z., Mo L., Zhou N., Chen W., Zhu H. (2025). Alzheimer’s Disease-Derived Outer Membrane Vesicles Exacerbate Cognitive Dysfunction, Modulate the Gut Microbiome, and Increase Neuroinflammation and Amyloid-β Production. Mol. Neurobiol..

[17] Gong T., Chen Q., Mao H., Zhang Y., Ren H., Xu M., Chen H., Yang D. (2022). Outer membrane vesicles of Porphyromonas gingivalis trigger NLRP3 inflammasome and induce neuroinflammation, tau phosphorylation, and memory dysfunction in mice. Front. Cell Infect. Microbiol..

[18] Sun B., Sawant H., Borthakur A., Bihl J.C. (2023). Emerging therapeutic role of gut microbial extracellular vesicles in neurological disorders. Front. Neurosci..

[19] Zhao X., Wei Y., Bu Y., Ren X., Dong Z. (2025). Review on bacterial outer membrane vesicles: Structure, vesicle formation, separation and biotechnological applications. Microb. Cell Fact..

[20] Magaña G., Harvey C., Taggart C.C., Rodgers A.M. (2023). Bacterial Outer Membrane Vesicles: Role in Pathogenesis and Host-Cell Interactions. Antibiotics.

[21] Furuyama N., Sircili M.P. (2021). Outer Membrane Vesicles (OMVs) Produced by Gram-Negative Bacteria: Structure, Functions, Biogenesis, and Vaccine Application. Biomed. Res. Int..

[22] Schwechheimer C., Kuehn M.J. (2015). Outer-membrane vesicles from Gram-negative bacteria: Biogenesis and functions. Nat. Rev. Microbiol..

[23] Jan A.T. (2017). Outer Membrane Vesicles (OMVs) of Gram-negative Bacteria: A Perspective Update. Front. Microbiol..

[24] Cecil J.D., Sirisaengtaksin N., O’Brien-Simpson N.M., Krachler A.M. (2019). Outer Membrane Vesicle-Host Cell Interactions. Microbiol. Spectr..

[25] Too L.K., Hunt N., Simunovic M.P. (2021). The Role of Inflammation and Infection in Age-Related Neurodegenerative Diseases: Lessons From Bacterial Meningitis Applied to Alzheimer Disease and Age-Related Macular Degeneration. Front. Cell Neurosci..

[26] Licastro F. (2022). Special Issue Editorial: “Infections, Inflammation and Neurodegeneration in Alzheimer Disease” Infections, Neuronal Senescence, and Dementia. Int. J. Mol. Sci..

[27] Shawkatova I., Durmanova V., Javor J. (2025). Alzheimer’s Disease and Porphyromonas gingivalis: Exploring the Links. Life.

[28] Huang Z., Hao M., Shi N., Wang X., Yuan L., Yuan H., Wang X. (2025). Porphyromonas gingivalis: A potential trigger of neurodegenerative disease. Front. Immunol..

[29] Sarmiento-Ordóñez J.M., Brito-Samaniego D.R., Vásquez-Palacios A.C., Pacheco-Quito E.M. (2025). Association Between Porphyromonas gingivalis and Alzheimer’s Disease: A Comprehensive Review. Infect. Drug Resist..

[30] Little C.S., Hammond C.J., MacIntyre A., Balin B.J., Appelt D.M. (2004). Chlamydia pneumoniae induces Alzheimer-like amyloid plaques in brains of BALB/c mice. Neurobiol. Aging.

[31] Garcia-Bustos V., Cabanero-Navalon M.D., Lloret-Sos C., Gómez M., Baquero M., Ibanez-Barcelo S., Grimaldos J., Cháfer-Pericás C. (2025). Exploring the role of multi-pathogen infections in Alzheimer’s disease: A case-control study. Virulence.

[32] Dourte M., Paître E., Bouchoucha M., Boyer E., Tomé S.O., Doeraene E., Huart C., Leroy K., Thal D.R., Decottignies A. (2025). The olfactory epithelium: A critical gateway for pathological tau propagation and a target for mitigating tauopathy in the central nervous system. Acta Neuropathol..

[33] Rajani V., Yuan Q. (2022). Noradrenergic Modulation of the Piriform Cortex: A Possible Avenue for Understanding Pre-Clinical Alzheimer’s Disease Pathogenesis. Front. Cell Neurosci..

[34] Liu Y., Cao Y., Wei H. (2025). Diagnostic value of olfactory function testing for Alzheimer’s disease and mild cognitive impairment: A systematic review and meta-analysis. Front. Aging Neurosci..

[35] Doty R.L. (2017). Olfactory dysfunction in neurodegenerative diseases: Is there a common pathological substrate?. Lancet Neurol..

[36] Buck L., Axel R. (1991). A novel multigene family may encode odorant receptors: A molecular basis for odor recognition. Cell.

[37] Wellford S.A., Moseman E.A. (2024). Olfactory immunology: The missing piece in airway and CNS defence. Nat. Rev. Immunol..

[38] Chen M., Reed R.R., Lane A.P. (2019). Chronic Inflammation Directs an Olfactory Stem Cell Functional Switch from Neuroregeneration to Immune Defense. Cell Stem Cell.

[39] Attems J., Walker L., Jellinger K.A. (2014). Olfactory bulb involvement in neurodegenerative diseases. Acta Neuropathol..

[40] Braak H., Braak E. (1991). Neuropathological stageing of Alzheimer-related changes. Acta Neuropathol..

[41] Bathini P., Brai E., Balin B.J., Bimler L., Corry D.B., Devanand D.P., Doty R.L., Ehrlich G.D., Eimer W.A., Fulop T. (2024). Sensory Dysfunction, Microbial Infections, and Host Responses in Alzheimer’s Disease. J. Infect. Dis..

[42] Cunha S., Forbes B., Sousa Lobo J.M., Silva A.C. (2021). Improving Drug Delivery for Alzheimer’s Disease Through Nose-to-Brain Delivery Using Nanoemulsions, Nanostructured Lipid Carriers (NLC) and in situ Hydrogels. Int. J. Nanomed..

[43] Kaisanlahti A., Salmi S., Kumpula S., Amatya S.B., Turunen J., Tejesvi M., Byts N., Tapiainen T., Reunanen J. (2023). Bacterial extracellular vesicles-brain invaders? A systematic review. Front. Mol. Neurosci..

[44] Dubé M., Le Coupanec A., Wong A.H.M., Rini J.M., Desforges M., Talbot P.J. (2018). Axonal Transport Enables Neuron-to-Neuron Propagation of Human Coronavirus OC43. J. Virol..

[45] Azzolino D., Carnevale-Schianca M., Santacroce L., Colella M., Felicetti A., Terranova L., Castrejón-Pérez R.C., Garcia-Godoy F., Lucchi T., Passarelli P.C. (2025). The Oral-Gut Microbiota Axis Across the Lifespan: New Insights on a Forgotten Interaction. Nutrients.

[46] Zhao D., Hu M., Liu S. (2024). Glial cells in the mammalian olfactory bulb. Front. Cell Neurosci..

[47] Devanand D.P., Lee S., Manly J., Andrews H., Schupf N., Doty R.L., Stern Y., Zahodne L.B., Louis E.D., Mayeux R. (2015). Olfactory deficits predict cognitive decline and Alzheimer dementia in an urban community. Neurology.

[48] Kettunen P., Koistinaho J., Rolova T. (2024). Contribution of CNS and extra-CNS infections to neurodegeneration: A narrative review. J. Neuroinflammation.

[49] Borrego-Ruiz A., Borrego J.J. (2025). Microbial Metabolomes in Alzheimer’s Disease: From Pathogenesis to Therapeutic Potential. Curr. Issues Mol. Biol..

[50] Lotz S.K., Blackhurst B.M., Reagin K.L., Funk K.E. (2021). Microbial Infections Are a Risk Factor for Neurodegenerative Diseases. Front. Cell Neurosci..

[51] Wang Y., Luo X., Xiang X., Hao C., Ma D. (2023). Roles of bacterial extracellular vesicles in systemic diseases. Front. Microbiol..

[52] Ha J.Y., Choi S.Y., Lee J.H., Hong S.H., Lee H.J. (2020). Delivery of Periodontopathogenic Extracellular Vesicles to Brain Monocytes and Microglial IL-6 Promotion by RNA Cargo. Front. Mol. Biosci..

[53] Chen S., Lei Q., Zou X., Ma D. (2023). The role and mechanisms of gram-negative bacterial outer membrane vesicles in inflammatory diseases. Front. Immunol..

[54] Pritchard A.B., Fabian Z., Lawrence C.L., Morton G., Crean S., Alder J.E. (2022). An Investigation into the Effects of Outer Membrane Vesicles and Lipopolysaccharide of Porphyromonas gingivalis on Blood-Brain Barrier Integrity, Permeability, and Disruption of Scaffolding Proteins in a Human in vitro Model. J. Alzheimers Dis..

[55] Wu Z., Long W., Yin Y., Tan B., Liu C., Li H., Ge S. (2025). Outer membrane vesicles of Porphyromonas gingivalis: Recent advances in pathogenicity and associated mechanisms. Front. Microbiol..

[56] Fatuzzo I., Niccolini G.F., Zoccali F., Cavalcanti L., Bellizzi M.G., Riccardi G., de Vincentiis M., Fiore M., Petrella C., Minni A. (2023). Neurons, Nose, and Neurodegenerative Diseases: Olfactory Function and Cognitive Impairment. Int. J. Mol. Sci..

[57] Liu D., Lu J., Wei L., Yao M., Yang H., Lv P., Wang H., Zhu Y., Zhu Z., Zhang X. (2024). Olfactory deficit: A potential functional marker across the Alzheimer’s disease continuum. Front. Neurosci..

[58] Coats S.R., Su T.H., Luderman Miller Z., King A.J., Ortiz J., Reddy A., Alaei S.R., Jain S. (2025). Porphyromonas gingivalis outer membrane vesicles divert host innate immunity and promote inflammation via C4′ monophosphorylated lipid A. J. Immunol..

[59] Carniglia L., Ramírez D., Durand D., Saba J., Caruso C., Lasaga M. (2016). [Nle4, D-Phe7]-α-MSH Inhibits Toll-Like Receptor (TLR)2- and TLR4-Induced Microglial Activation and Promotes a M2-Like Phenotype. PLoS ONE.

[60] Xing J., McKenzie T., Hu J. (2025). Lipid-Laden Microglia: Characterization and Roles in Diseases. Cells.

[61] Ridler C. (2017). Neuroimmunology: Microglia-induced reactive astrocytes-toxic players in neurological disease?. Nat. Rev. Neurol..

[62] Zamanian J.L., Xu L., Foo L.C., Nouri N., Zhou L., Giffard R.G., Barres B.A. (2012). Genomic analysis of reactive astrogliosis. J. Neurosci..

[63] Muramoto S., Shimizu S., Shirakawa S., Ikeda H., Miyamoto S., Jo M., Takemori U., Morimoto C., Wu Z., Tozaki-Saitoh H. (2025). Noradrenaline Synergistically Enhances Porphyromonas gingivalis LPS and OMV-Induced Interleukin-1β Production in BV-2 Microglia Through Differential Mechanisms. Int. J. Mol. Sci..

[64] Palacios E., Lobos-González L., Guerrero S., Kogan M.J., Shao B., Heinecke J.W., Quest A.F.G., Leyton L., Valenzuela-Valderrama M. (2023). Helicobacter pylori outer membrane vesicles induce astrocyte reactivity through nuclear factor-κappa B activation and cause neuronal damage in vivo in a murine model. J. Neuroinflammation.

[65] Calì C., Cantando I., Veloz Castillo M.F., Gonzalez L., Bezzi P. (2024). Metabolic Reprogramming of Astrocytes in Pathological Conditions: Implications for Neurodegenerative Diseases. Int. J. Mol. Sci..

[66] Charpentier L.A., Dolben E.F., Hendricks M.R., Hogan D.A., Bomberger J.M., Stanton B.A. (2023). Bacterial Outer Membrane Vesicles and Immune Modulation of the Host. Membranes.

[67] Miao J., Chen L., Pan X., Li L., Zhao B., Lan J. (2023). Microglial Metabolic Reprogramming: Emerging Insights and Therapeutic Strategies in Neurodegenerative Diseases. Cell Mol. Neurobiol..

[68] Habib N., McCabe C., Medina S., Varshavsky M., Kitsberg D., Dvir-Szternfeld R., Green G., Dionne D., Nguyen L., Marshall J.L. (2020). Disease-associated astrocytes in Alzheimer’s disease and aging. Nat. Neurosci..

[69] Li C., Peng J., Xiao L., Wu H., Chen J., Chen N. (2025). Pseudomonas aeruginosa-derived extracellular vesicles enhance macrophage aerobic glycolysis that fuels inflammation. Front. Microbiol..

[70] Zhang Q., Wang S.S., Zhang Z., Chu S.F. (2025). PKM2-mediated metabolic reprogramming of microglia in neuroinflammation. Cell Death Discov..

[71] Sapkota A., Halder S.K., Milner R. (2025). Blood-brain barrier disruption and microglial activation during hypoxia and post-hypoxic recovery in aged mice. Brain Commun..

[72] Nie R., Wu Z., Ni J., Zeng F., Yu W., Zhang Y., Kadowaki T., Kashiwazaki H., Teeling J.L., Zhou Y. (2019). Porphyromonas gingivalis Infection Induces Amyloid-β Accumulation in Monocytes/Macrophages. J. Alzheimers Dis..

[73] Catalan E.A., Seguel-Fuentes E., Fuentes B., Aranguiz-Varela F., Castillo-Godoy D.P., Rivera-Asin E., Bocaz E., Fuentes J.A., Bravo D., Schinnerling K. (2024). Oral Pathobiont-Derived Outer Membrane Vesicles in the Oral-Gut Axis. Int. J. Mol. Sci..

[74] Dominy S.S., Lynch C., Ermini F., Benedyk M., Marczyk A., Konradi A., Nguyen M., Haditsch U., Raha D., Griffin C. (2019). Porphyromonas gingivalis in Alzheimer’s disease brains: Evidence for disease causation and treatment with small-molecule inhibitors. Sci. Adv..

[75] Fan Z., Tang P., Li C., Yang Q., Xu Y., Su C., Li L. (2023). Fusobacterium nucleatum and its associated systemic diseases: Epidemiologic studies and possible mechanisms. J. Oral. Microbiol..

[76] Zhang L., Zhang D., Liu C., Tang B., Cui Y., Guo D., Duan M., Tu Y., Zheng H., Ning X. (2024). Outer Membrane Vesicles Derived From Fusobacterium nucleatum Trigger Periodontitis Through Host Overimmunity. Adv. Sci..

[77] Cuesta C.M., Guerri C., Ureña J., Pascual M. (2021). Role of Microbiota-Derived Extracellular Vesicles in Gut-Brain Communication. Int. J. Mol. Sci..

[78] Meng D., Lai Y., Zhang L., Hu W., Wei H., Guo C., Jing X., Zhou H., Xiao R., Zhu L. (2024). Helicobacter pylori outer membrane vesicles directly promote Aβ aggregation and enhance Aβ toxicity in APP/PS1 mice. Commun. Biol..

[79] Socorro Ruiz-Palma M.D., Avila-Calderón E.D., Aguilera-Arreola M.G., López-Merino A., Ruiz E.A., Morales-García M.D.R., López-Villegas E.O., Gomez-Lunar Z., Arellano-Reynoso B., Contreras-Rodríguez A. (2021). Comparative proteomic analysis of outer membrane vesicles from Brucella suis, Brucella ovis, Brucella canis and Brucella neotomae. Arch. Microbiol..

[80] Alaei S.R., King A.J., Banani K., Reddy A., Ortiz J., Knight A.L., Haldeman J., Su T.H., Park H., Coats S.R. (2025). Lipid a remodeling modulates outer membrane vesicle biogenesis by Porphyromonas gingivalis. J. Bacteriol..

[81] Hill D.J., Virji M. (2003). A novel cell-binding mechanism of Moraxella catarrhalis ubiquitous surface protein UspA: Specific targeting of the N-domain of carcinoembryonic antigen-related cell adhesion molecules by UspA1. Mol. Microbiol..

[82] Perez Vidakovics M.L., Riesbeck K. (2009). Virulence mechanisms of Moraxella in the pathogenesis of infection. Curr. Opin. Infect. Dis..

[83] Bullard B., Lipski S.L., Lafontaine E.R. (2005). Hag directly mediates the adherence of Moraxella catarrhalis to human middle ear cells. Infect. Immun..

[84] Hill D.J., Whittles C., Virji M. (2012). A novel group of Moraxella catarrhalis UspA proteins mediates cellular adhesion via CEACAMs and vitronectin. PLoS ONE.

[85] Singh B., Al-Jubair T., Voraganti C., Andersson T., Mukherjee O., Su Y.C., Zipfel P., Riesbeck K. (2015). Moraxella catarrhalis Binds Plasminogen To Evade Host Innate Immunity. Infect. Immun..

[86] Su Y.C., Kadari M., Straw M.L., Janoušková M., Jonsson S., Thofte O., Jalalvand F., Matuschek E., Sandblad L., Végvári Á. (2023). Non-typeable Haemophilus influenzae major outer membrane protein P5 contributes to bacterial membrane stability, and affects the membrane protein composition crucial for interactions with the human host. Front. Cell Infect. Microbiol..

[87] Zariri A., Beskers J., van de Waterbeemd B., Hamstra H.J., Bindels T.H., van Riet E., van Putten J.P., van der Ley P. (2016). Meningococcal Outer Membrane Vesicle Composition-Dependent Activation of the Innate Immune Response. Infect. Immun..

[88] Ho M.H., Chen C.H., Goodwin J.S., Wang B.Y., Xie H. (2015). Functional Advantages of Porphyromonas gingivalis Vesicles. PLoS ONE.

[89] Mantri C.K., Chen C.H., Dong X., Goodwin J.S., Pratap S., Paromov V., Xie H. (2015). Fimbriae-mediated outer membrane vesicle production and invasion of Porphyromonas gingivalis. Microbiologyopen.

[90] Nichols F.C., Clark R.B., Maciejewski M.W., Provatas A.A., Balsbaugh J.L., Dewhirst F.E., Smith M.B., Rahmlow A. (2020). A novel phosphoglycerol serine-glycine lipodipeptide of Porphyromonas gingivalis is a TLR2 ligand. J. Lipid Res..

[91] Wielento A., Bereta G.P., Łagosz-Ćwik K.B., Eick S., Lamont R.J., Grabiec A.M., Potempa J. (2022). TLR2 Activation by Porphyromonas gingivalis Requires Both PPAD Activity and Fimbriae. Front. Immunol..

[92] Liang S., Krauss J.L., Domon H., McIntosh M.L., Hosur K.B., Qu H., Li F., Tzekou A., Lambris J.D., Hajishengallis G. (2011). The C5a receptor impairs IL-12-dependent clearance of Porphyromonas gingivalis and is required for induction of periodontal bone loss. J. Immunol..

[93] Blasi F., Centanni S., Allegra L. (2004). Chlamydia pneumoniae: Crossing the barriers?. Eur. Respir. J..

[94] Tcw J., Qian L., Pipalia N.H., Chao M.J., Liang S.A., Shi Y., Jain B.R., Bertelsen S.E., Kapoor M., Marcora E. (2022). Cholesterol and matrisome pathways dysregulated in astrocytes and microglia. Cell.

[95] Tsang V.S.K., Malaspina A., Henson S.M. (2025). The metabolic intersection between immunosenescence and neuroinflammation in amyotrophic lateral sclerosis. J. Inflamm..

[96] Ge J., Cao M., Zhang Y., Wu T., Liu J., Pu J., He H., Guo Z., Ju S., Yu J. (2025). Inhibiting NLRP3 enhances cellular autophagy induced by outer membrane vesicles from Pseudomonas aeruginosa. Microbiol. Spectr..

[97] Bakleh M.Z., Al Haj Zen A. (2025). The Distinct Role of HIF-1α and HIF-2α in Hypoxia and Angiogenesis. Cells.

[98] Tang Y.Y., Wang D.C., Wang Y.Q., Huang A.F., Xu W.D. (2022). Emerging role of hypoxia-inducible factor-1α in inflammatory autoimmune diseases: A comprehensive review. Front. Immunol..

[99] van Gisbergen M.W., Offermans K., Voets A.M., Lieuwes N.G., Biemans R., Hoffmann R.F., Dubois L.J., Lambin P. (2020). Mitochondrial Dysfunction Inhibits Hypoxia-Induced HIF-1α Stabilization and Expression of Its Downstream Targets. Front. Oncol..

[100] Mesentier-Louro L.A., Rangel B., Stell L., Shariati M.A., Dalal R., Nathan A., Yuan K., de Jesus Perez V., Liao Y.J. (2021). Hypoxia-induced inflammation: Profiling the first 24-hour posthypoxic plasma and central nervous system changes. PLoS ONE.

[101] Yusri K., Jose S., Vermeulen K.S., Tan T.C.M., Sorrentino V. (2025). The role of NAD(+) metabolism and its modulation of mitochondria in aging and disease. npj Metab. Health Dis..

[102] Chen W., Wu Y., Liang Y., Su X., Ke M., Deng D., Zang J., Zhu J., Mai H., Xu A. (2025). Small Extracellular Vesicles From Hypoxia-Neuron Maintain Blood-Brain Barrier Integrity. Stroke.

[103] Banks W.A., Sharma P., Bullock K.M., Hansen K.M., Ludwig N., Whiteside T.L. (2020). Transport of Extracellular Vesicles across the Blood-Brain Barrier: Brain Pharmacokinetics and Effects of Inflammation. Int. J. Mol. Sci..

[104] Ramos-Zaldívar H.M., Polakovicova I., Salas-Huenuleo E., Corvalán A.H., Kogan M.J., Yefi C.P., Andia M.E. (2022). Extracellular vesicles through the blood-brain barrier: A review. Fluids Barriers CNS.

[105] Díaz-Garrido N., Badia J., Baldomà L. (2021). Microbiota-derived extracellular vesicles in interkingdom communication in the gut. J. Extracell. Vesicles.

[106] Xia X., Fang Y., Zhong J., Li F., Jiang L. (2025). Biomimetic strategies of cell membrane vesicles driven by pathogen-host interactions: Novel insights into antimicrobial immunotherapy and infection prevention. Front. Immunol..

[107] Felicetti A., Azzolino D., Piro P.P., Lopes G.C.D., Rezaeinezhad N., Lovero R., Bocchio-Chiavetto L., Colella M., Passarelli P.C. (2025). The Oral-Brain Axis in Alzheimer’s Disease: From Microbial Dysbiosis to Neurodegeneration. Microorganisms.

[108] Narengaowa, Kong W., Lan F., Awan U.F., Qing H., Ni J. (2021). The Oral-Gut-Brain AXIS: The Influence of Microbes in Alzheimer’s Disease. Front. Cell Neurosci..

[109] Schaar V., Nordström T., Mörgelin M., Riesbeck K. (2011). Moraxella catarrhalis outer membrane vesicles carry β-lactamase and promote survival of Streptococcus pneumoniae and Haemophilus influenzae by inactivating amoxicillin. Antimicrob. Agents Chemother..

[110] Augustyniak D., Seredyński R., McClean S., Roszkowiak J., Roszniowski B., Smith D.L., Drulis-Kawa Z., Mackiewicz P. (2018). Virulence factors of Moraxella catarrhalis outer membrane vesicles are major targets for cross-reactive antibodies and have adapted during evolution. Sci. Rep..

[111] Vidakovics M.L., Jendholm J., Mörgelin M., Månsson A., Larsson C., Cardell L.O., Riesbeck K. (2010). B cell activation by outer membrane vesicles—A novel virulence mechanism. PLoS Pathog..

[112] Thakur S., Dhapola R., Sarma P., Medhi B., Reddy D.H. (2023). Neuroinflammation in Alzheimer’s Disease: Current Progress in Molecular Signaling and Therapeutics. Inflammation.

[113] Schubert-Unkmeir A., Konrad C., Slanina H., Czapek F., Hebling S., Frosch M. (2010). Neisseria meningitidis induces brain microvascular endothelial cell detachment from the matrix and cleavage of occludin: A role for MMP-8. PLoS Pathog..

[114] Borkowski J., Li L., Steinmann U., Quednau N., Stump-Guthier C., Weiss C., Findeisen P., Gretz N., Ishikawa H., Tenenbaum T. (2014). Neisseria meningitidis elicits a pro-inflammatory response involving IκBζ in a human blood-cerebrospinal fluid barrier model. J. Neuroinflammation.

[115] Olsen I. (2021). Porphyromonas gingivalis-Induced Neuroinflammation in Alzheimer’s Disease. Front. Neurosci..

[116] Chacko A., Delbaz A., Walkden H., Basu S., Armitage C.W., Eindorf T., Trim L.K., Miller E., West N.P., St John J.A. (2022). Chlamydia pneumoniae can infect the central nervous system via the olfactory and trigeminal nerves and contributes to Alzheimer’s disease risk. Sci. Rep..

[117] Gérard H.C., Dreses-Werringloer U., Wildt K.S., Deka S., Oszust C., Balin B.J., Frey W.H., Bordayo E.Z., Whittum-Hudson J.A., Hudson A.P. (2006). Chlamydophila (Chlamydia) pneumoniae in the Alzheimer’s brain. FEMS Immunol. Med. Microbiol..

[118] Romanella A., McCall M., Corwin R., Faruq A.A., Lingo E., Bhambhani S., Hammond C.J., Balin B.J. (2025). Infections with Chlamydia pneumoniae and SARS-CoV-2 and Alzheimer’s disease pathogenesis. Front. Aging Neurosci..

[119] O’Donoghue E.J., Krachler A.M. (2016). Mechanisms of outer membrane vesicle entry into host cells. Cell Microbiol..

[120] Yáñez-Mó M., Siljander P.R., Andreu Z., Zavec A.B., Borràs F.E., Buzas E.I., Buzas K., Casal E., Cappello F., Carvalho J. (2015). Biological properties of extracellular vesicles and their physiological functions. J. Extracell. Vesicles.

[121] David L., Taieb F., Pénary M., Bordignon P.J., Planès R., Bagayoko S., Duplan-Eche V., Meunier E., Oswald E. (2022). Outer membrane vesicles produced by pathogenic strains of Escherichia coli block autophagic flux and exacerbate inflammasome activation. Autophagy.

[122] He Y., Shiotsu N., Uchida-Fukuhara Y., Guo J., Weng Y., Ikegame M., Wang Z., Ono K., Kamioka H., Torii Y. (2020). Outer membrane vesicles derived from Porphyromonas gingivalis induced cell death with disruption of tight junctions in human lung epithelial cells. Arch. Oral. Biol..

[123] Krsek D., Yara D.A., Hrbáčková H., Daniel O., Mančíková A., Schüller S., Bielaszewska M. (2023). Translocation of outer membrane vesicles from enterohemorrhagic Escherichia coli O157 across the intestinal epithelial barrier. Front. Microbiol..

[124] Jones E.J., Booth C., Fonseca S., Parker A., Cross K., Miquel-Clopés A., Hautefort I., Mayer U., Wileman T., Stentz R. (2020). The Uptake, Trafficking, and Biodistribution of Bacteroides thetaiotaomicron Generated Outer Membrane Vesicles. Front. Microbiol..

[125] Zhang Y., Wang H., Zhang Y., Zhao P., Li Y. (2023). Aerosolization inhalation of non-typeable Haemophilus influenzae outer membrane vesicles contributing to neutrophilic asthma. Front. Microbiol..

[126] Mekata M., Yoshida K., Takai A., Hiroshima Y., Ikuta A., Seyama M., Yoshida K., Ozaki K. (2025). Porphyromonas gingivalis outer membrane vesicles increase vascular permeability by inducing stress fiber formation and degrading vascular endothelial-cadherin in endothelial cells. FEBS J..

[127] Zhang Z., Liu D., Liu S., Zhang S., Pan Y. (2020). The Role of Porphyromonas gingivalis Outer Membrane Vesicles in Periodontal Disease and Related Systemic Diseases. Front. Cell Infect. Microbiol..

[128] Miyashita N., Matsumoto A., Friedman H., Yamamoto Y., Bendinelli M. (2004). Morphology of Chlamydia pneumoniae. Chlamydia pneumoniae Infection and Disease.

[129] Mussa F.F., Chai H., Wang X., Yao Q., Lumsden A.B., Chen C. (2006). Chlamydia pneumoniae and vascular disease: An update. J. Vasc. Surg..

[130] Neyra Chauca J.M., Robles Martinez G.G. (2025). Histological and Functional Breakdown of the Blood-Brain Barrier in Alzheimer’s Disease: A Multifactorial Intersection. Neurol. Int..

[131] Sharpe S.W., Kuehn M.J., Mason K.M. (2011). Elicitation of epithelial cell-derived immune effectors by outer membrane vesicles of nontypeable Haemophilus influenzae. Infect. Immun..

[132] Nonaka S., Kadowaki T., Nakanishi H. (2022). Secreted gingipains from Porphyromonas gingivalis increase permeability in human cerebral microvascular endothelial cells through intracellular degradation of tight junction proteins. Neurochem. Int..

[133] Kim J.H., Yoon Y.J., Lee J., Choi E.J., Yi N., Park K.S., Park J., Lötvall J., Kim Y.K., Gho Y.S. (2013). Outer membrane vesicles derived from Escherichia coli up-regulate expression of endothelial cell adhesion molecules in vitro and in vivo. PLoS ONE.

[134] Pérez-Cremades D., Bueno-Betí C., García-Giménez J.L., Ibañez-Cabellos J.S., Pallardó F.V., Hermenegildo C., Novella S. (2023). Extracellular histones trigger oxidative stress-dependent induction of the NF-kB/CAM pathway via TLR4 in endothelial cells. J. Physiol. Biochem..

[135] Kaparakis-Liaskos M., Ferrero R.L. (2015). Immune modulation by bacterial outer membrane vesicles. Nat. Rev. Immunol..

[136] Park J.Y., Park H.M., Kim S., Jeon K.B., Lim C.M., Hong J.T., Yoon D.Y. (2023). Human IL-32θA94V mutant attenuates monocyte-endothelial adhesion by suppressing the expression of ICAM-1 and VCAM-1 via binding to cell surface receptor integrin αVβ3 and αVβ6 in TNF-α-stimulated HUVECs. Front. Immunol..

[137] Denaro S., D’Aprile S., Alberghina C., Pavone A.M., Torrisi F., Giallongo S., Longhitano L., Mannino G., Lo Furno D., Zappalà A. (2022). Neurotrophic and immunomodulatory effects of olfactory ensheathing cells as a strategy for neuroprotection and regeneration. Front. Immunol..

[138] Zhang L.P., Liao J.X., Liu Y.Y., Luo H.L., Zhang W.J. (2023). Potential therapeutic effect of olfactory ensheathing cells in neurological diseases: Neurodegenerative diseases and peripheral nerve injuries. Front. Immunol..

[139] Xia B., Gao J., Li S., Huang L., Ma T., Zhao L., Yang Y., Huang J., Luo Z. (2019). Extracellular Vesicles Derived From Olfactory Ensheathing Cells Promote Peripheral Nerve Regeneration in Rats. Front. Cell Neurosci..

[140] LaFever B.J., Imamura F. (2022). Effects of nasal inflammation on the olfactory bulb. J. Neuroinflammation.

[141] Ahmed A.A.Q., Besio R., Xiao L., Forlino A. (2023). Outer Membrane Vesicles (OMVs) as Biomedical Tools and Their Relevance as Immune-Modulating Agents against H. pylori Infections: Current Status and Future Prospects. Int. J. Mol. Sci..

[142] Chaulagain B., Gothwal A., Lamptey R.N.L., Trivedi R., Mahanta A.K., Layek B., Singh J. (2023). Experimental Models of In Vitro Blood-Brain Barrier for CNS Drug Delivery: An Evolutionary Perspective. Int. J. Mol. Sci..

[143] Conners R., Hill D.J., Borodina E., Agnew C., Daniell S.J., Burton N.M., Sessions R.B., Clarke A.R., Catto L.E., Lammie D. (2008). The Moraxella adhesin UspA1 binds to its human CEACAM1 receptor by a deformable trimeric coiled-coil. EMBO J..

[144] Brooks M.J., Sedillo J.L., Wagner N., Wang W., Attia A.S., Wong H., Laurence C.A., Hansen E.J., Gray-Owen S.D. (2008). Moraxella catarrhalis binding to host cellular receptors is mediated by sequence-specific determinants not conserved among all UspA1 protein variants. Infect. Immun..

[145] Frohlich K.M., Hua Z., Quayle A.J., Wang J., Lewis M.E., Chou C.W., Luo M., Buckner L.R., Shen L. (2014). Membrane vesicle production by Chlamydia trachomatis as an adaptive response. Front. Cell Infect. Microbiol..

[146] Bhatnagar S., Shinagawa K., Castellino F.J., Schorey J.S. (2007). Exosomes released from macrophages infected with intracellular pathogens stimulate a proinflammatory response in vitro and in vivo. Blood.

[147] Soria F.N., Pampliega O., Bourdenx M., Meissner W.G., Bezard E., Dehay B. (2017). Exosomes, an Unmasked Culprit in Neurodegenerative Diseases. Front. Neurosci..

[148] Noh M.Y., Kwon H.S., Kwon M.S., Nahm M., Jin H.K., Bae J.S., Kim S.H. (2025). Biomarkers and therapeutic strategies targeting microglia in neurodegenerative diseases: Current status and future directions. Mol. Neurodegener..

[149] Rangel-Gomez M., Alberini C.M., Deneen B., Drummond G.T., Manninen T., Sur M., Vicentic A. (2024). Neuron-Glial Interactions: Implications for Plasticity, Behavior, and Cognition. J. Neurosci..

[150] Fiebich B.L., Batista C.R.A., Saliba S.W., Yousif N.M., de Oliveira A.C.P. (2018). Role of Microglia TLRs in Neurodegeneration. Front. Cell Neurosci..

[151] Irrera N., Russo M., Pallio G., Bitto A., Mannino F., Minutoli L., Altavilla D., Squadrito F. (2020). The Role of NLRP3 Inflammasome in the Pathogenesis of Traumatic Brain Injury. Int. J. Mol. Sci..

[152] Kienes I., Weidl T., Mirza N., Chamaillard M., Kufer T.A. (2021). Role of NLRs in the Regulation of Type I Interferon Signaling, Host Defense and Tolerance to Inflammation. Int. J. Mol. Sci..

[153] Peignier A., Parker D. (2021). Impact of Type I Interferons on Susceptibility to Bacterial Pathogens. Trends Microbiol..

[154] Madrer N., Perera N.D., Uccelli N.A., Abbondanza A., Andersen J.V., Carsana E.V., Demmings M.D., Fernandez R.F., de Fragas M.G., Gbadamosi I. (2025). Neural Metabolic Networks: Key Elements of Healthy Brain Function. J. Neurochem..

[155] Zlokovic B.V. (2011). Neurovascular pathways to neurodegeneration in Alzheimer’s disease and other disorders. Nat. Rev. Neurosci..

[156] Cheung G., Sibille J., Zapata J., Rouach N. (2015). Activity-Dependent Plasticity of Astroglial Potassium and Glutamate Clearance. Neural Plast..

[157] Gharbi T., Zhang Z., Yang G.Y. (2020). The Function of Astrocyte Mediated Extracellular Vesicles in Central Nervous System Diseases. Front. Cell Dev. Biol..

[158] Lauro C., Limatola C. (2020). Metabolic Reprograming of Microglia in the Regulation of the Innate Inflammatory Response. Front. Immunol..

[159] Cabrera-Pastor A. (2024). Extracellular Vesicles as Mediators of Neuroinflammation in Intercellular and Inter-Organ Crosstalk. Int. J. Mol. Sci..

[160] Zhang Y.M., Qi Y.B., Gao Y.N., Chen W.G., Zhou T., Zang Y., Li J. (2023). Astrocyte metabolism and signaling pathways in the CNS. Front. Neurosci..

[161] Thangwong P., Tocharus C., Tocharus J. (2025). The Bidirectional Role of Hypoxia-Inducible Factor 1 Alpha in Vascular Dementia Caused by Chronic Cerebral Hypoperfusion. Mol. Neurobiol..

[162] Belaidi A.A., Bush A.I., Ayton S. (2025). Apolipoprotein E in Alzheimer’s disease: Molecular insights and therapeutic opportunities. Mol. Neurodegener..

[163] Toyofuku M., Schild S., Kaparakis-Liaskos M., Eberl L. (2023). Composition and functions of bacterial membrane vesicles. Nat. Rev. Microbiol..

[164] Yang L.G., March Z.M., Stephenson R.A., Narayan P.S. (2023). Apolipoprotein E in lipid metabolism and neurodegenerative disease. Trends Endocrinol. Metab..

[165] Wang N., Wang M., Jeevaratnam S., Rosenberg C., Ikezu T.C., Shue F., Doss S.V., Alnobani A., Martens Y.A., Wren M. (2022). Opposing effects of apoE2 and apoE4 on microglial activation and lipid metabolism in response to demyelination. Mol. Neurodegener..

[166] Victor M.B., Leary N., Luna X., Meharena H.S., Scannail A.N., Bozzelli P.L., Samaan G., Murdock M.H., von Maydell D., Effenberger A.H. (2022). Lipid accumulation induced by APOE4 impairs microglial surveillance of neuronal-network activity. Cell Stem Cell.

[167] Haney M.S., Pálovics R., Munson C.N., Long C., Johansson P.K., Yip O., Dong W., Rawat E., West E., Schlachetzki J.C.M. (2024). APOE4/4 is linked to damaging lipid droplets in Alzheimer’s disease microglia. Nature.

[168] Chien T.Y., Chiang C.S. (2025). Activated astrocytes drive the accumulation of apolipoprotein E at the brain tumor edge. Brain Tumor Pathol..

[169] Lindner K., Beckenbauer K., van Ek L.C., Titeca K., de Leeuw S.M., Awwad K., Hanke F., Korepanova A.V., Rybin V., van der Kam E.L. (2022). Isoform- and cell-state-specific lipidation of ApoE in astrocytes. Cell Rep..

[170] Mukherjee S., Suresh S.N. (2019). Neuron-Astrocyte Liaison to Maintain Lipid Metabolism of Brain. Trends Endocrinol. Metab..

[171] Chen H., Zhao S., Jian Q., Yan Y., Wang S., Zhang X., Ji Y. (2024). The role of ApoE in fatty acid transport from neurons to astrocytes under ischemia/hypoxia conditions. Mol. Biol. Rep..

[172] Nolt G.L., Golden L.R., Thorpe S.P., Funnell J.L., Stephens I.O., Hernandez G., MacLean S.M., Lucido C.C., Brock C.R., Pallerla A.V. (2025). Microglia-derived APOE2 improves remyelination even in the presence of endogenous APOE4. J. Neuroinflammation.

[173] Loving B.A., Bruce K.D. (2020). Lipid and Lipoprotein Metabolism in Microglia. Front. Physiol..

[174] Dose J., Huebbe P., Nebel A., Rimbach G. (2016). APOE genotype and stress response—A mini review. Lipids Health Dis..

[175] Grimaldi L., Bovi E., Formisano R., Sancesario G. (2024). ApoE: The Non-Protagonist Actor in Neurological Diseases. Genes.

[176] Yassine H.N., Hugo C., O’Donovan B., Stephens I.O., Johnson L.A., Cole G., Tcw J., Johansson J., Kisler K., Chiba-Falek O. (2025). APOE-Targeted Therapeutics for Alzheimer’s Disease. J. Neurosci..

[177] Rajič Bumber J., Rački V., Mežnarić S., Pelčić G., Mršić-Pelčić J. (2025). Clinical Significance of APOE4 Genotyping: Potential for Personalized Therapy and Early Diagnosis of Alzheimer’s Disease. J. Clin. Med..

[178] Pérez M., Avila J., Hernández F. (2019). Propagation of Tau via Extracellular Vesicles. Front. Neurosci..

[179] Fowler S.L., Behr T.S., Turkes E., O’Brien D.P., Cauhy P.M., Rawlinson I., Edmonds M., Foiani M.S., Schaler A., Crowley G. (2025). Tau filaments are tethered within brain extracellular vesicles in Alzheimer’s disease. Nat. Neurosci..

[180] Preeti K., Sood A., Fernandes V. (2022). Metabolic Regulation of Glia and Their Neuroinflammatory Role in Alzheimer’s Disease. Cell Mol. Neurobiol..

[181] McGroarty J., Salinas S., Evans H., Jimenez B., Tran V., Kadavakollu S., Vashist A., Atluri V. (2025). Inflammasome-Mediated Neuroinflammation: A Key Driver in Alzheimer’s Disease Pathogenesis. Biomolecules.

[182] Shen Y., Wang X., Liu X., Wang G., Hou X., Zhou X. (2025). Research Progress of Lipid Metabolism-Mediated Neuroinflammation in Alzheimer’s Disease. Cell Mol. Neurobiol..

[183] Leroux E., Perbet R., Caillierez R., Richetin K., Lieger S., Espourteille J., Bouillet T., Bégard S., Danis C., Loyens A. (2022). Extracellular vesicles: Major actors of heterogeneity in tau spreading among human tauopathies. Mol. Ther..

[184] Hicks D.A., Nalivaeva N.N., Turner A.J. (2012). Lipid rafts and Alzheimer’s disease: Protein-lipid interactions and perturbation of signaling. Front. Physiol..

[185] Kim H.S., Kim S., Shin S.J., Park Y.H., Nam Y., Kim C.W., Lee K.W., Kim S.M., Jung I.D., Yang H.D. (2021). Gram-negative bacteria and their lipopolysaccharides in Alzheimer’s disease: Pathologic roles and therapeutic implications. Transl. Neurodegener..

[186] Urano Y., Hayashi I., Isoo N., Reid P.C., Shibasaki Y., Noguchi N., Tomita T., Iwatsubo T., Hamakubo T., Kodama T. (2005). Association of active gamma-secretase complex with lipid rafts. J. Lipid Res..

[187] Tiku V., Tan M.W. (2021). Host immunity and cellular responses to bacterial outer membrane vesicles. Trends Immunol..

[188] Chow J.C., Young D.W., Golenbock D.T., Christ W.J., Gusovsky F. (1999). Toll-like receptor-4 mediates lipopolysaccharide-induced signal transduction. J. Biol. Chem..

[189] Prakash P., Manchanda P., Paouri E., Bisht K., Sharma K., Rajpoot J., Wendt V., Hossain A., Wijewardhane P.R., Randolph C.E. (2025). Amyloid-β induces lipid droplet-mediated microglial dysfunction via the enzyme DGAT2 in Alzheimer’s disease. Immunity.

[190] Wu X., Miller J.A., Lee B.T.K., Wang Y., Ruedl C. (2025). Reducing microglial lipid load enhances β amyloid phagocytosis in an Alzheimer’s disease mouse model. Sci. Adv..

[191] Zhang H., Jin Q., Li J., Wang J., Li M., Yin Q., Li Q., Qi Y., Feng L., Shen L. (2025). Astrocyte-derived complement C3 facilitated microglial phagocytosis of synapses in Staphylococcus aureus-associated neurocognitive deficits. PLoS Pathog..

[192] Raposo G., Stoorvogel W. (2013). Extracellular vesicles: Exosomes, microvesicles, and friends. J. Cell Biol..

[193] Kulp A., Kuehn M.J. (2010). Biological functions and biogenesis of secreted bacterial outer membrane vesicles. Annu. Rev. Microbiol..

[194] Coughlan C., Lindenberger J., Jacot J.G., Johnson N.R., Anton P., Bevers S., Welty R., Graner M.W., Potter H. (2024). Specific Binding of Alzheimer’s Aβ Peptides to Extracellular Vesicles. Int. J. Mol. Sci..

[195] Srivastava A.K., Pittman J.M., Zerweck J., Venkata B.S., Moore P.C., Sachleben J.R., Meredith S.C. (2019). β-Amyloid aggregation and heterogeneous nucleation. Protein Sci..

[196] Prosswimmer T., Heng A., Daggett V. (2024). Mechanistic insights into the role of amyloid-β in innate immunity. Sci. Rep..

[197] Diamond J.S. (2005). Deriving the glutamate clearance time course from transporter currents in CA1 hippocampal astrocytes: Transmitter uptake gets faster during development. J. Neurosci..

[198] D’Mello S.R. (2021). When Good Kinases Go Rogue: GSK3, p38 MAPK and CDKs as Therapeutic Targets for Alzheimer’s and Huntington’s Disease. Int. J. Mol. Sci..

[199] Yu H., Xiong M., Zhang Z. (2023). The role of glycogen synthase kinase 3 beta in neurodegenerative diseases. Front. Mol. Neurosci..

[200] Yoon S.O., Park D.J., Ryu J.C., Ozer H.G., Tep C., Shin Y.J., Lim T.H., Pastorino L., Kunwar A.J., Walton J.C. (2012). JNK3 perpetuates metabolic stress induced by Aβ peptides. Neuron.

[201] Zhang Y., Tan J., Miao Y., Zhang Q. (2021). The effect of extracellular vesicles on the regulation of mitochondria under hypoxia. Cell Death Dis..

[202] Sontag E., Nunbhakdi-Craig V., Lee G., Bloom G.S., Mumby M.C. (1996). Regulation of the phosphorylation state and microtubule-binding activity of Tau by protein phosphatase 2A. Neuron.

[203] Tang J., Huang H., Muirhead R.C.J., Zhou Y., Li J., DeFelice J., Kopanitsa M.V., Serneels L., Davey K., Tilley B.S. (2024). Associations of amyloid-β oligomers and plaques with neuropathology in the App (NL-G-F) mouse. Brain Commun..

[204] Meng X., Song Q., Liu Z., Liu X., Wang Y., Liu J. (2024). Neurotoxic β-amyloid oligomers cause mitochondrial dysfunction-the trigger for PANoptosis in neurons. Front. Aging Neurosci..

[205] Zyśk M., Beretta C., Naia L., Dakhel A., Påvénius L., Brismar H., Lindskog M., Ankarcrona M., Erlandsson A. (2023). Amyloid-β accumulation in human astrocytes induces mitochondrial disruption and changed energy metabolism. J. Neuroinflammation.

[206] Pérez M.J., Jara C., Quintanilla R.A. (2018). Contribution of Tau Pathology to Mitochondrial Impairment in Neurodegeneration. Front. Neurosci..

[207] Kulkarni A., Jozefiaková J., Bhide K., Mochnaćová E., Bhide M. (2023). Differential transcriptome response of blood brain barrier spheroids to neuroinvasive Neisseria and Borrelia. Front. Cell Infect. Microbiol..

[208] Schubert-Unkmeir A. (2017). Molecular mechanisms involved in the interaction of Neisseria meningitidis with cells of the human blood-cerebrospinal fluid barrier. Pathog. Dis..

[209] Falcicchia C., Tozzi F., Gabrielli M., Amoretti S., Masini G., Nardi G., Guglielmo S., Ratto G.M., Arancio O., Verderio C. (2023). Microglial extracellular vesicles induce Alzheimer’s disease-related cortico-hippocampal network dysfunction. Brain Commun..

[210] Zhang S., Crossley C.A., Yuan Q. (2024). Neuronal Vulnerability of the Entorhinal Cortex to Tau Pathology in Alzheimer’s Disease. Br. J. Biomed. Sci..

[211] Cyr B., Cabrera Ranaldi E., Hadad R., Dietrich W.D., Keane R.W., de Rivero Vaccari J.P. (2024). Extracellular vesicles mediate inflammasome signaling in the brain and heart of Alzheimer’s disease mice. Front. Mol. Neurosci..

[212] Anselmo S., Bonaccorso E., Gangemi C., Sancataldo G., Conti Nibali V., D’Angelo G. (2025). Lipid Rafts in Signalling, Diseases, and Infections: What Can Be Learned from Fluorescence Techniques?. Membranes.

[213] Zevini A., Olagnier D., Hiscott J. (2017). Crosstalk between Cytoplasmic RIG-I and STING Sensing Pathways. Trends Immunol..

[214] Decout A., Katz J.D., Venkatraman S., Ablasser A. (2021). The cGAS-STING pathway as a therapeutic target in inflammatory diseases. Nat. Rev. Immunol..

[215] Nazmi A., Field R.H., Griffin E.W., Haugh O., Hennessy E., Cox D., Reis R., Tortorelli L., Murray C.L., Lopez-Rodriguez A.B. (2019). Chronic neurodegeneration induces type I interferon synthesis via STING, shaping microglial phenotype and accelerating disease progression. Glia.

[216] Reynolds M.B., Klein B., McFadden M.J., Judge N.K., Navarrete H.E., Michmerhuizen B.C., Awad D., Schultz T.L., Harms P.W., Zhang L. (2024). Type I interferon governs immunometabolic checkpoints that coordinate inflammation during Staphylococcal infection. Cell Rep..

[217] Ahmadi Badi S., Bruno S.P., Moshiri A., Tarashi S., Siadat S.D., Masotti A. (2020). Small RNAs in Outer Membrane Vesicles and Their Function in Host-Microbe Interactions. Front. Microbiol..

[218] Cosacak M.I., Bhattarai P., Kizil C. (2020). Alzheimer’s disease, neural stem cells and neurogenesis: Cellular phase at single-cell level. Neural Regen. Res..

[219] Roveta F., Bonino L., Piella E.M., Rainero I., Rubino E. (2024). Neuroinflammatory Biomarkers in Alzheimer’s Disease: From Pathophysiology to Clinical Implications. Int. J. Mol. Sci..

[220] Qiu M., Li J., Wu W., Ren J., Wu X. (2025). The dual role of type I interferons in bacterial infections: From immune defense to pathogenesis. mBio.

[221] Kumar A., Su Y., Sharma M., Singh S., Kim S., Peavey J.J., Suerken C.K., Lockhart S.N., Whitlow C.T., Craft S. (2023). MicroRNA expression in extracellular vesicles as a novel blood-based biomarker for Alzheimer’s disease. Alzheimers Dement..

[222] Szabo L., Eckert A., Grimm A. (2020). Insights into Disease-Associated Tau Impact on Mitochondria. Int. J. Mol. Sci..

[223] Bitto N.J., Chapman R., Pidot S., Costin A., Lo C., Choi J., D’Cruze T., Reynolds E.C., Dashper S.G., Turnbull L. (2017). Bacterial membrane vesicles transport their DNA cargo into host cells. Sci. Rep..

[224] Haas-Neill S., Forsythe P. (2020). A Budding Relationship: Bacterial Extracellular Vesicles in the Microbiota-Gut-Brain Axis. Int. J. Mol. Sci..

[225] Olsen I., Singhrao S.K. (2015). Can oral infection be a risk factor for Alzheimer’s disease?. J. Oral. Microbiol..

[226] Elhabbari K., Sireci S., Rothermel M., Brunert D. (2024). Olfactory deficits in aging and Alzheimer’s-spotlight on inhibitory interneurons. Front. Neurosci..

[227] Mulet M., Sánchez Milán J.A., Lorca C., Fernández-Rhodes M., Adrados-Planell A., Bejarano Castillo M.C., Saiz L., Mateos-Moreno M.V., Hase Y., Mira A. (2025). Oral Microbiome-Derived Proteins in Brain Extracellular Vesicles Circulate and Tie to Specific Dysbiotic and Neuropathological Profiles in Age-Related Dementias. Mol. Cell Proteom..

[228] Mallach A., Zielonka M., van Lieshout V., An Y., Khoo J.H., Vanheusden M., Chen W.T., Moechars D., Arancibia-Carcamo I.L., Fiers M. (2024). Microglia-astrocyte crosstalk in the amyloid plaque niche of an Alzheimer’s disease mouse model, as revealed by spatial transcriptomics. Cell Rep..

[229] Mancini F., Rossi O., Necchi F., Micoli F. (2020). OMV Vaccines and the Role of TLR Agonists in Immune Response. Int. J. Mol. Sci..

[230] Supplie L.M., Düking T., Campbell G., Diaz F., Moraes C.T., Götz M., Hamprecht B., Boretius S., Mahad D., Nave K.A. (2017). Respiration-Deficient Astrocytes Survive As Glycolytic Cells In Vivo. J. Neurosci..

[231] Gao C., Jiang J., Tan Y., Chen S. (2023). Microglia in neurodegenerative diseases: Mechanism and potential therapeutic targets. Signal Transduct. Target. Ther..

[232] Sun Y., Wei K., Liao X., Wang J., Gao L., Pang B. (2025). Lipid metabolism in microglia: Emerging mechanisms and therapeutic opportunities for neurodegenerative diseases (Review). Int. J. Mol. Med..

[233] Han X., Wang C., Song L., Wang X., Tang S., Hou T., Liu C., Liang X., Qiu C., Wang Y. (2022). KIBRA regulates amyloid β metabolism by controlling extracellular vesicles secretion. EBioMedicine.

[234] Tracy T.E., Madero-Pérez J., Swaney D.L., Chang T.S., Moritz M., Konrad C., Ward M.E., Stevenson E., Hüttenhain R., Kauwe G. (2022). Tau interactome maps synaptic and mitochondrial processes associated with neurodegeneration. Cell.

[235] Ehehalt R., Keller P., Haass C., Thiele C., Simons K. (2003). Amyloidogenic processing of the Alzheimer beta-amyloid precursor protein depends on lipid rafts. J. Cell Biol..

[236] Banerjee S., Hashemi M., Zagorski K., Lyubchenko Y.L. (2021). Cholesterol in Membranes Facilitates Aggregation of Amyloid β Protein at Physiologically Relevant Concentrations. ACS Chem. Neurosci..

[237] Miyake K., Shibata T., Fukui R., Murakami Y., Sato R., Hiranuma R. (2024). Endosomal Toll-Like Receptors as Therapeutic Targets for Autoimmune Diseases. Adv. Exp. Med. Biol..

[238] Silbereis J.C., Pochareddy S., Zhu Y., Li M., Sestan N. (2016). The Cellular and Molecular Landscapes of the Developing Human Central Nervous System. Neuron.

[239] Takei N., Nawa H. (2014). mTOR signaling and its roles in normal and abnormal brain development. Front. Mol. Neurosci..

[240] Han J., Zhang X., Kang L., Guan J. (2025). Extracellular vesicles as therapeutic modulators of neuroinflammation in Alzheimer’s disease: A focus on signaling mechanisms. J. Neuroinflammation.

[241] Ghosh M., Bayat A.H., Pearse D.D. (2025). Small Extracellular Vesicles in Neurodegenerative Disease: Emerging Roles in Pathogenesis, Biomarker Discovery, and Therapy. Int. J. Mol. Sci..

[242] Kapogiannis D., Mustapic M., Shardell M.D., Berkowitz S.T., Diehl T.C., Spangler R.D., Tran J., Lazaropoulos M.P., Chawla S., Gulyani S. (2019). Association of Extracellular Vesicle Biomarkers With Alzheimer Disease in the Baltimore Longitudinal Study of Aging. JAMA Neurol..

[243] Zhang X., Wijenayake S., Hossain S., Liu Q. (2025). Estimating progression of Alzheimer’s disease with extracellular vesicle-related multi-omics risk models. Front. Aging Neurosci..

[244] Jack C.R., Andrews S.J., Beach T.G., Buracchio T., Dunn B., Graf A., Hansson O., Ho C., Jagust W., McDade E. (2024). Revised criteria for the diagnosis and staging of Alzheimer’s disease. Nat. Med..

[245] Hampel H., Hu Y., Cummings J., Mattke S., Iwatsubo T., Nakamura A., Vellas B., O’Bryant S., Shaw L.M., Cho M. (2023). Blood-based biomarkers for Alzheimer’s disease: Current state and future use in a transformed global healthcare landscape. Neuron.

[246] Rajendran K., Krishnan U.M. (2024). Biomarkers in Alzheimer’s disease. Clin. Chim. Acta.

[247] Lin C., Tian Y., Tanzi R.E., Jorfi M. (2024). Approaches for studying neuroimmune interactions in Alzheimer’s disease. Trends Immunol..

[248] Lee H.S., Boulton I.C., Reddin K., Wong H., Halliwell D., Mandelboim O., Gorringe A.R., Gray-Owen S.D. (2007). Neisserial outer membrane vesicles bind the coinhibitory receptor carcinoembryonic antigen-related cellular adhesion molecule 1 and suppress CD4+ T lymphocyte function. Infect. Immun..

[249] Gerritzen M.J.H., Maas R.H.W., van den Ijssel J., van Keulen L., Martens D.E., Wijffels R.H., Stork M. (2018). High dissolved oxygen tension triggers outer membrane vesicle formation by Neisseria meningitidis. Microb. Cell Fact..

[250] Yu Y., Wang Z., Chai Z., Ma S., Li A., Li Y. (2025). Central Nervous System-Derived Extracellular Vesicles as Biomarkers in Alzheimer’s Disease. Int. J. Mol. Sci..

[251] Dai Y., Cui Y., Li J., Li P., Huang X. (2025). Integrated microfluidic platforms for extracellular vesicles: Separation, detection, and clinical translation. APL Bioeng..

[252] Lee S., Mankhong S., Kang J.H. (2019). Extracellular Vesicle as a Source of Alzheimer’s Biomarkers: Opportunities and Challenges. Int. J. Mol. Sci..

[253] Preciado D., Poley M., Tsai S., Tomney A., Brown K., Val S. (2016). A proteomic characterization of NTHi lysates. Int. J. Pediatr. Otorhinolaryngol..

[254] Khan A., Raza F., He N. (2024). Nanoscale Extracellular Vesicle-Enabled Liquid Biopsy: Advances and Challenges for Lung Cancer Detection. Micromachines.

[255] Lara B., Sassot M., Calo G., Paparini D., Gliosca L., Chaufan G., Loureiro I., Vota D., Ramhorst R., Pérez Leirós C. (2023). Extracellular Vesicles of Porphyromonas gingivalis Disrupt Trophoblast Cell Interaction with Vascular and Immune Cells in an In Vitro Model of Early Placentation. Life.

[256] Butola M., Nainwal N. (2024). Non-Invasive Techniques of Nose to Brain Delivery Using Nanoparticulate Carriers: Hopes and Hurdles. AAPS PharmSciTech.

[257] Couch Y. (2023). Challenges associated with using extracellular vesicles as biomarkers in neurodegenerative disease. Expert. Rev. Mol. Diagn..

[258] Chen J., Tian C., Xiong X., Yang Y., Zhang J. (2025). Extracellular vesicles: New horizons in neurodegeneration. EBioMedicine.

[259] Dhapola R., Kumari S., Sharma P., Paidlewar M., Medhi B., Vellingiri B., HariKrishnaReddy D. (2025). Deciphering Molecular and Signaling Pathways of Extracellular Vesicles-Based Therapeutics for Alzheimer’s Disease. Mol. Neurobiol..

[260] Pacoova Dal Maschio V., Roveta F., Bonino L., Boschi S., Rainero I., Rubino E. (2025). The Role of Blood-Based Biomarkers in Transforming Alzheimer’s Disease Research and Clinical Management: A Review. Int. J. Mol. Sci..

[261] Serna M.F., Mosquera M., García-Perdomo H.A. (2025). Inflammatory Markers and their Relationship with Cognitive Function in Alzheimer’s Disease and Mild Cognitive Impairment. Systematic Review and Meta-Analysis. Neuromolecular Med..

[262] de Leeuw D.M., Trieu C., Vromen E.M., Blujdea E.R., Verberk I.M.W., Duits F.H., Teunissen C.E., Pijnenburg Y.A.L., van der Flier W.M., van Harten A.C. (2025). Cerebrospinal Fluid Amyloid and Tau Biomarker Changes Across the Alzheimer Disease Clinical Spectrum. JAMA Netw. Open.

[263] Cacciaglia R., Shekari M., Salvadó G., Milà-Alomà M., Falcon C., Sánchez-Benavides G., Minguillón C., Fauria K., Grau-Rivera O., Molinuevo J.L. (2025). The CSF p-tau/β-amyloid 42 ratio correlates with brain structure and fibrillary β-amyloid deposition in cognitively unimpaired individuals at the earliest stages of pre-clinical Alzheimer’s disease. Brain Commun..

[264] Wang Z., Chen Y., Gong K., Zhao B., Ning Y., Chen M., Li Y., Ali M., Timsina J., Liu M. (2025). Cerebrospinal fluid proteomics identification of biomarkers for amyloid and tau PET stages. Cell Rep. Med..

[265] Farmen K., Tofiño-Vian M., Iovino F. (2021). Neuronal Damage and Neuroinflammation, a Bridge Between Bacterial Meningitis and Neurodegenerative Diseases. Front. Cell Neurosci..

[266] Mozaheb N., Mingeot-Leclercq M.P. (2020). Membrane Vesicle Production as a Bacterial Defense Against Stress. Front. Microbiol..

[267] Li X., Chen J., Yang Y., Cai H., Ao Z., Xing Y., Li K., Yang K., Guan W., Friend J. (2025). Extracellular vesicle-based point-of-care testing for diagnosis and monitoring of Alzheimer’s disease. Microsyst. Nanoeng..

[268] Gu L., Meng R., Tang Y., Zhao K., Liang F., Zhang R., Xue Q., Chen F., Xiao X., Wang H. (2019). Toll-Like Receptor 4 Signaling Licenses the Cytosolic Transport of Lipopolysaccharide From Bacterial Outer Membrane Vesicles. Shock.

[269] Pedrioli G., Paganetti P. (2020). Hijacking Endocytosis and Autophagy in Extracellular Vesicle Communication: Where the Inside Meets the Outside. Front. Cell Dev. Biol..

[270] Ozkocak D.C., Phan T.K., Poon I.K.H. (2022). Translating extracellular vesicle packaging into therapeutic applications. Front. Immunol..

[271] Chen Z., Xiong M., Tian J., Song D., Duan S., Zhang L. (2024). Encapsulation and assessment of therapeutic cargo in engineered exosomes: A systematic review. J. Nanobiotechnol..

[272] Megha, London E. (2004). Ceramide selectively displaces cholesterol from ordered lipid domains (rafts): Implications for lipid raft structure and function. J. Biol. Chem..

[273] Rodriguez B.V., Kuehn M.J. (2020). Staphylococcus aureus secretes immunomodulatory RNA and DNA via membrane vesicles. Sci. Rep..

[274] Xu S., Chaudhary O., Rodríguez-Morales P., Sun X., Chen D., Zappasodi R., Xu Z., Pinto A.F.M., Williams A., Schulze I. (2021). Uptake of oxidized lipids by the scavenger receptor CD36 promotes lipid peroxidation and dysfunction in CD8(+) T cells in tumors. Immunity.

[275] Negah S.S., Moradi H.R., Forouzanfar F., Sahraian M.A., Faraji M. (2025). The Role of Small Extracellular Vesicles Derived from Glial Cells in the Central Nervous System under both Normal and Pathological Conditions. Neurochem. Res..

[276] Yu M., Ma H., Lai X., Wu J., Shen M., Yan J. (2025). Stem cell extracellular vesicles: A new dawn for anti-inflammatory treatment of neurodegenerative diseases. Front. Aging Neurosci..

[277] Pistono C., Bister N., Stanová I., Malm T. (2020). Glia-Derived Extracellular Vesicles: Role in Central Nervous System Communication in Health and Disease. Front. Cell Dev. Biol..

[278] Yao J., Irwin R.W., Zhao L., Nilsen J., Hamilton R.T., Brinton R.D. (2009). Mitochondrial bioenergetic deficit precedes Alzheimer’s pathology in female mouse model of Alzheimer’s disease. Proc. Natl. Acad. Sci. USA.

[279] Verdin E. (2015). NAD^+^ in aging, metabolism, and neurodegeneration. Science.

[280] Rubio-Atonal L.F., Ioannou M.S. (2023). Astrocytic OxPhos: More than just energy production. Nat. Metab..

[281] Rose J., Brian C., Pappa A., Panayiotidis M.I., Franco R. (2020). Mitochondrial Metabolism in Astrocytes Regulates Brain Bioenergetics, Neurotransmission and Redox Balance. Front. Neurosci..

[282] Afridi R., Lee W.H., Suk K. (2020). Microglia Gone Awry: Linking Immunometabolism to Neurodegeneration. Front. Cell Neurosci..

[283] Dong J., Gao Z., Liu M., Qian B., Yuan C., Liu H., Rao N., Liu Y. (2025). Astrocyte-Neuron Metabolic Synergies in Neurological Homeostasis and Disease. Neurochem. Res..

[284] Bonvento G., Bolaños J.P. (2021). Astrocyte-neuron metabolic cooperation shapes brain activity. Cell Metab..

[285] Sarazin M., Lagarde J., El Haddad I., de Souza L.C., Bellier B., Potier M.C., Bottlaender M., Dorothée G. (2024). The path to next-generation disease-modifying immunomodulatory combination therapies in Alzheimer’s disease. Nat. Aging.

[286] Singh S., Shukla R. (2023). Nanovesicular-Mediated Intranasal Drug Therapy for Neurodegenerative Disease. AAPS PharmSciTech.

[287] Brożek-Mądry E., Ziuzia-Januszewska L., Misztal O., Burska Z., Sosnowska-Turek E., Sierdziński J. (2025). Nasal Rinsing with Probiotics-Microbiome Evaluation in Patients with Inflammatory Diseases of the Nasal Mucosa. J. Clin. Med..

[288] Al-Romaih S., Harati O., Mfuna L.E., Filali-Mouhim A., Pelletier A., Renteria Flores A., Desrosiers M. (2023). Response to intranasal Lactococcus lactis W136 probiotic supplementation in refractory CRS is associated with modulation of non-type 2 inflammation and epithelial regeneration. Front. Allergy.

[289] Xu M., Ren M., Zhang X., Peng W., Li H., Liao W., Xie J., Zhang X. (2025). Mesenchymal stem cell-derived small extracellular vesicles restored nasal barrier function in allergic rhinitis via miR-143-GSK3B in human nasal epithelial cells. J. Allergy Clin. Immunol..

